# Promotion of DFU Wound Healing via BRG1–COL16A1 Axis in Fibroblasts

**DOI:** 10.1002/advs.202516687

**Published:** 2026-03-02

**Authors:** Penghui Wang, Jiaming Sun, Ying Wang, Bichen Ren, Yuxin Liu, Yunhan Liu, Liang Chen, Lu Yu, Tao Dai, Li Yu, Zhihui Dong

**Affiliations:** ^1^ Department of Vascular Surgery Institute of Vascular Surgery Zhongshan Hospital Fudan University Shanghai China; ^2^ Department of Plastic & Reconstructive Surgery Shanghai Ninth People's Hospital Shanghai Jiao Tong University School of Medicine Shanghai China; ^3^ Children's Medical Center School of Medicine Shanghai Jiao Tong University Shanghai China; ^4^ State Key Laboratory of Genetic Engineering School of Life Science Fudan University Shanghai China; ^5^ Department of Wound Reconstructive Surgery Tongji Hospital of Tongji University Shanghai China

**Keywords:** BRG1, COL16A1, DFU, Fibroblast, Wound Repair

## Abstract

Diabetic foot ulceration (DFU) imposes major global health burdens due to high morbidity and recurrence rates. Current therapies (debridement and offloading) show limited efficacy, highlighting the need to uncover molecular mechanisms of diabetic wound repair. Multi‐omics analyses of DFU and healthy skin identify fibroblast‐derived COL16A1 as a wound healing mediator. In vitro, COL16A1 overexpression or knockdown in fibroblasts is employed to assess its regulatory effects on cellular function and collagen secretory capacity. In vivo, localized adenoviral delivery of COL16A1 is administered to evaluate its therapeutic impact on wound closure in diabetic mice. DNA pull‐down plus LC‐MS/MS, ChIP‐qPCR, and luciferase reporter assays are systematically implemented to delineate the upstream regulatory mechanism of COL16A1 expression. Transcriptomic profiling of skin specimens identifies fibroblast‐derived COL16A1 as a key regulatory gene in DFU repair. Subsequent in vitro and in vivo experiments demonstrate COL16A1 enhances fibroblast activation and restores normal repair processes in diabetic wounds. Notably, Brahma‐related gene 1 (BRG1) is identified as the key transcription factor governing COL16A1 expression. BRG1 directly binds to the COL16A1 promoter, upregulating its transcription and thereby accelerating diabetic wound healing. BRG1‐COL16A1 axis is a critical regulatory pathway and proposes novel therapeutic targets for chronic diabetic wounds.

## Graphical abstract

In normal wound healing, transcription factor BRG1 is upregulated and binds the COL16A1 promoter to enhance its expression, promoting fibroblast proliferation, migration, contraction, extracellular matrix deposition, and granulation tissue formation, thus accelerating wound closure. In diabetic wounds, this BRG1‐COL16A1 axis is impaired, delaying healing, suggesting targeted modulation of this pathway as a potential therapy for diabetic wound repair.

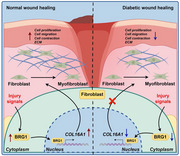

**Abbreviations**
DFU: Diabetic foot ulceration
scRNA‐seq: single‐cell RNA sequencing
BRG1: Brahma‐related gene 1
ECM: extracellular matrix
FACIT: fibril‐associated collagens with interrupted triple helices
SWI/SNF: switch/sucrose non‐fermentable
ROS: reactive oxygen species
DEGs: differentially expressed genes
GO: Gene Ontology
PPI: Protein‐protein interaction

## 1 Introduction

Diabetic foot ulceration (DFU), a prevalent long‐term diabetic complication, leads to lower extremity amputation in ∼20% of cases [1, 2]. Patients with DFU exhibit a 5‐year mortality rate of 30%, which escalates to over 70% among those undergoing above‐foot amputation [3]. Lower extremity amputations are predominantly caused by delayed wound healing, secondary infections, and progressive gangrene in patients with diabetes [4]. The orchestrated healing process involves sequential phases encompassing clot formation (hemostasis), inflammatory recruitment, proliferative tissue repair (keratinocyte migration and fibroblast‐driven granulation), and extracellular matrix (ECM) remodeling, mediated by interactions among epithelial, stromal, and immune cells [5]. Unlike acute wounds, DFU fails to progress through normal healing phases owing to a detrimental triad of hyperglycemia‐induced cellular dysfunction, neuro‐ischemic compromise, and oxidative stress from reactive oxygen species (ROS) accumulation. Although numerous therapeutic approaches for DFU are clinically available, including standard debridement [6], offloading [7, 8, 9], and multiple wound dressings [10, 11], 23% of patients exhibit persistent non‐healing DFU at 12 months post‐diagnosis, with recurrence rates following therapeutic intervention reaching 42% and 65% at 1‐ and 5‐years follow‐up intervals, respectively [12]. Therefore, elucidating the cellular and molecular mechanisms underlying DFU wound healing is of paramount importance.

Fibroblasts, a primary cellular component of the skin, participate in multiple phases of wound healing. During tissue repair, these cells are activated, migrate to the wound bed, demonstrate contractile properties, and deposit ECM onto granulation tissue, thereby filling tissue defects and facilitating wound closure [13]. COL16A1, a member of the fibril‐associated collagens with interrupted triple helices (FACIT) family, is a minor collagen component in connective tissues [14]. In the skin, COL16A1 is predominantly synthesized by dermal fibroblasts, smooth muscle cells, and dendritic cells [15, 16], predominantly localized to the dermal‐epidermal junction and superficial dermis rather than the deep dermis [17]. The current understanding of COL16A1 expression regulation remains limited, with only a few studies reporting TGFβ2‐mediated COL16A1 mRNA level upregulation in dermal fibroblasts. Previous research suggests COL16A1 may function as a structural component within the microfibrillar apparatus of the skin, potentially stabilizing interactions between microfibrils and other matrix components [18]. However, COL16A1 role in wound healing processes remains unclear.

Brahma‐related gene 1 (BRG1, encoded by SMARCA4) is the core enzymatic component of the switch/sucrose non‐fermentable (SWI/SNF) chromatin remodeling complex. It plays a pivotal role in modulating chromatin architecture, ultimately mediating transcriptional activation or repression [19]. This molecular machinery is fundamentally involved in regulating DNA repair [20], gene expression [21], redox homeostasis [22], and cell cycle progression [23]. Emerging evidence indicates that BRG1 enhances cutaneous wound re‐epithelialization by modulating keratinocyte migratory dynamics [24]. Furthermore, BRG1 orchestrates NF‐κB recruitment to transcriptionally activate Sonic Hedgehog in hair matrix cells, thereby maintaining hair follicle regeneration. This molecular axis concurrently stimulates tissue repair by enhancing progenitor cell activation [25]. It remains unclear whether BRG1 regulates COL16A1 expression.

Our investigation employed single‐cell sequencing and transcriptomic profiling to identify COL16A1 in fibroblasts as a pivotal regulator of DFU healing. Functional validation was performed to substantiate its role in wound healing, both in vitro and in vivo. Subsequent mechanistic studies using ChIP‐qPCR revealed a novel BRG1‐COL16A1 regulatory axis, wherein BRG1 mediated the COL16A1 transcriptional upregulation to potentiate fibroblast functional activation and drove tissue regeneration cascades, thus proposing a novel therapeutic paradigm for refractory diabetic wound healing.

## 2 Material and Methods

### 2.1 Sample Collection

Patients with DFU and non‐diabetic healthy controls were recruited from Zhongshan Hospital, Fudan University. DFU skin samples (referred as DFU)were obtained from patients with diabetes during lower extremity amputations, whereas healthy control skin specimens (referred as CTRL) were collected from non‐diabetic individuals undergoing foot surgeries, such as hallux valgus correction or traumatic injury repair. Discarded foot skin specimens were collected during clinically indicated procedures and processed for analysis. This study adhered to the principles outlined in the Declaration of *Helsinki* and was approved by the Medical Ethics Committee of Zhongshan Hospital, Fudan University (Approval No.: B2022‐488R2). Written informed consent was obtained from all participants.

### 2.2 Animal

Male C57BL/6J (8 weeks) and male C57BL/6J‐db/db (8 weeks) mice were procured from Jiangsu Huachuang Sino (Jiangsu, China). All animal studies, including the euthanasia procedure for mice, were conducted following the regulations and guidelines established by the Animal Experiment and Care Committee of Shanghai Ninth People's Hospital, Shanghai Jiaotong University School of Medicine (Approval No.: SH9H‐2025‐A1586‐1).

### 2.3 Excisional Wound Healing Model

Following anesthesia with Zoletil‐50 and dexmedetomidine (80/0.75 mg/kg), 8–10 week‐old C57BL/6 and db/db mice underwent dorsal hair removal and antisepsis with 70% ethanol. Two full‐thickness 8 mm excisional wounds penetrating both the skin and panniculus carnosus muscle were created on the dorsum using a sterile biopsy punch (KAI, Japan) [26]. Wounds were photographed at defined intervals (post‐wound day (PWD) 0, 2, 4, 6, and 8) under standardized conditions, and wound areas were quantified using ImageJ software. The percentage of wound area was calculated as follows: percentage of wound area (%) = Timepoint area/Day 0 area × 100. At designated endpoints (days 2, 4, 6, and 8), mice were euthanized, and tissue specimens comprising the wound bed with a 2‐mm peripheral margin were excised for subsequent analysis.

### 2.4 scRNA‐Seq analyses

Skin specimens were maintained in sterile PBS on ice and processed within 3 h post‐excision. Tissue samples were dissected into 0.5 mm^3^ fragments and rinsed extensively with PBS to remove adventitious tissues (for example, blood clots and adipose layers). The tissues were then digested and filtered to get single‐cell suspensions. Overall cell viability was confirmed using trypan blue exclusion, which was above 85%. Single‐cell suspensions were loaded onto a 10x Chromium Controller (10x Genomics) targeting 5000 cells per sample following the protocol of the manufacturer (Chromium Single Cell 3′ Reagent Kits v3). cDNA amplification and library construction were performed according to standard procedures. Libraries were sequenced on an Illumina NovaSeq 6000 platform (LC‐Bio Technology Co., Hangzhou) with 150‐bp paired‐end reads at a minimum depth of 20000 reads per cell.

### 2.5 RNA Sequencing and Differentially Expressed Gene Analysis

Total RNA was isolated from skin tissues of CTRL and DFU groups using TRIzol reagent (Solarbio, China). RNA sequencing and primary bioinformatics processing were performed by Kangchen Bio‐tech (Shanghai, China). Differential gene expression analysis was conducted using the DESeq2 package in R, with significantly differentially expressed genes (DEGs) defined by |log_2_FC| > 0.585 and adjusted *p* value < 0.005.

### 2.6 Adenovirus Vector Packaging and Transfection

Recombinant adenoviral vectors (Ad) encoding COL16A1, BRG1 (SMARCA4), and empty vector controls (Ad‐NC) were packaged by Genomeditech (Shanghai, China). Primary mouse dermal fibroblasts were transduced at multiplicities of infection of 10 (Ad‐OE‐COL16A1) and 25 (Ad‐OE‐BRG1), respectively. To evaluate therapeutic effects on wound healing, adenoviral suspensions in 50 µL 0.9% saline Ad‐OE‐COL16A1 (2 × 10^7^ PFU), Ad‐OE‐BRG1 (1 × 10^8^ PFU), and Ad‐NC, were administered via intradermal injection at peri‐wound sites on the dorsal skin. Injections were administered 3 days before full‐thickness excisional wound creation.

### 2.7 siRNA Transfection

Primary mouse dermal fibroblasts were transfected with gene‐specific siRNAs to silence target genes using Lipofectamine 3000 reagent (#L3000150, Thermo Fisher Scientific) according to the protocol of the manufacturer. Negative control siRNAs and validated targeting siRNAs were obtained from GenePharma Co., Ltd. (Shanghai, China). Cells were incubated with siRNA‐Lipofectamine complexes for 4 days before functional assays, and knockdown efficiency was confirmed by RT‐qPCR/Western blot.

### 2.8 Primary Mouse Dermal Fibroblast Isolation and Culture

Primary fibroblasts were isolated from neonatal C57BL/6 mice (postnatal days 1–3). Following cervical dislocation and 20 min sterilization in 75% ethanol, the dorsolateral skin was aseptically excised. The subcutaneous fat and vasculature were meticulously removed under sterile conditions. Tissues were minced into < 1 mm^3^ fragments and digested in 0.1% collagenase type I (Amsbio) at 37°C for 1 h with gentle agitation. Digestion was terminated by adding an equal volume of Dulbecco's modified Eagle's medium (DMEM, 5.5 mmol/L glucose) supplemented with 10% FBS (Sigma) and 1% penicillin‐streptomycin, referred to as low‐glucose (LG) medium. The suspension was centrifuged at 1000 rpm/min for 5 min. After supernatant removal, pellets were resuspended in PBS and centrifuged again. Tissue explants were transferred to T25 flasks containing LG medium and incubated at 37°C/5% CO_2_ with medium changes every 72 h. At 80–90% confluence, cells were passaged and reseeded at a 1:3 ratio in LG medium. Fibroblasts between passages 3–8 were used for experiments. The high‐glucose (HG) medium consisted of DMEM (25 mmol/L glucose) supplemented with 10% FBS and 1% penicillin‐streptomycin. Cells were cultured in either LG or HG medium for four days before the experiment.

### 2.9 Cell Viability Assays

Cell viability was assessed using a Cell Counting Kit‐8 (CCK‐8; Dojindo, Japan), same as our perious study [27]. Briefly, primary mouse dermal fibroblasts were seeded in 96‐well plates at 8 × 10^3^ cells/well density. The cells were incubated at 37°C for 2 h in medium with 10% CCK‐8 reagent. Optical density was quantified at 450 nm using a microplate reader (Thermo, USA).

### 2.10 EdU Cell Proliferation Assay

Primary mouse dermal fibroblasts were seeded in 96‐well plates at 8 × 10^3^ cells/well density. After 24 h of culture, cell proliferation was evaluated using the BeyoClick EdU‐488 Kit (#C0071S, Beyotime). Cells were incubated with 10 µM EdU for 2 h at 37°C, fixed with 4% paraformaldehyde (15 min), permeabilized with 0.3% Triton X‐100 (20 min), and subsequently reacted with Click Reaction Cocktail for 1 h (light protected). The nuclei were counterstained with Hoechst 33342 (12 h, light protected). Images were captured using an Axio Vert.A1 microscope (Zeiss, Oberkochen, Germany), and the proliferation rates calculated as: EdU^+^ cells (%) = (Number of EdU^+^ nuclei/Total nuclei) × 100.

### 2.11 Transwell Migration Assay

The cells were serum‐starved in 0.5% FBS/DMEM for 12 h, harvested with 0.25% trypsin‐EDTA, and resuspended in serum‐free medium. Cells (1 × 10^5^; in 200 µL serum‐free DMEM) were seeded into uncoated Transwell inserts (24‐well, 8 µm pore; #3422, Corning) placed over lower chambers containing 600 µL DMEM with 20% FBS chemoattractant. After 6 h incubation (37°C/5% CO_2_), non‐migrated cells were removed from the upper membrane surface using a cotton swab. The migrated cells were fixed with 4% PFA (15 min), stained with 0.1% crystal violet (20 min), and quantified by phase‐contrast microscopy.

### 2.12 Cell Contraction Assay

Rat Tail Collagen Type I (#354236, Corning) was combined with sterile 5 × PBS (diluted to 1 × final concentration) and 0.1 M NaOH on ice to prepare the neutralized collagen working solution. The mixture was gently mixed to achieve a homogeneous, bubble‐free solution at physiological pH (∼7.4) and isotonicity, targeting a final collagen concentration of 1 mg/mL. Seed suspended cells (2 × 10^5^/well) were immediately added to the neutralized collagen mixture, pipetted into 24‐well plates, and incubated at 37°C for 20 min to facilitate polymerization. Following solidification, the gels were carefully released by overlaying with culture medium, contraction was monitored over 6 h, and gel area reduction as a cellular contractility measure was quantified.

### 2.13 RNA Purification and Quantitative Real‐Time PCR (RT‐qPCR)

Total RNA was extracted using TRIzol reagent (Solarbio, China). Reverse transcription was performed using a PrimeScript RT reagent kit (Takara, Japan). RT‐qPCR was subsequently performed on a QuantStudio 6 Flex system (Thermo Fisher Scientific, USA) using SYBR Green qPCR Master Mix (Vazyme, China) according to the protocols of the manufacturers. Gene expression levels were quantified using the ΔΔCT method with GAPDH as the endogenous control. All mRNA primer sequences are listed in Tables S1 and S2.

### 2.14 Western Blot Assay

Tissues and cultured cells were lysed for 30 min in RIPA buffer (Solarbio) supplemented with 1 mM PMSF according to the protocol of the manufacturer. Protein samples (20 µg per lane) were separated by SDS‐PAGE and electrophoretically transferred to PVDF membranes (Millipore). After blocking with 5% skim milk for 1 h at room temperature (RT), membranes were incubated overnight at 4°C with the following primary antibodies: Anti‐GAPDH (10494‐1‐AP, Proteintech; 1:10,000), anti‐COL16A1 (ab231044, abcam; 1:1000), anti‐BRG1 (66561‐1‐Ig, Proteintech; 1:1000), anti‐COL1A1 (86093‐1‐RR, Proteintech, 1:1000), anti‐COL3A1 (22734‐1‐AP, Proteintech, 1:1000), anti‐⍺SMA (67735‐1‐Ig, Proteintech; 1:1000), and anti‐TGFβ1 (ab315254, abcam; 1:1000). Next day, membranes were incubated with HRP‐conjugated secondary antibodies for 1 h at RT. Protein bands were visualized using enhanced chemiluminescence substrate and detected using an Amersham Imager 600 (GE Healthcare, USA).

### 2.15 Hematoxylin and Eosin (H&E) Staining

Tissues were fixed overnight in 4% paraformaldehyde and paraffin‐embedded, and sectioned at 5 µm thickness. Following deparaffinization and rehydration, sections were stained with modified H&E, respectively. Wound morphology histological analysis was performed using a Nikon Eclipse light microscope.

### 2.16 Masson's Trichrome Staining

Paraformaldehyde‐fixed paraffin‐embedded tissues were sectioned at 5 µm, deparaffinized, and rehydrated. The sections underwent sequential staining with Weigert's hematoxylin (5 min), Biebrich scarlet‐acid fuchsin (5 min), phosphotungstic/phosphomolybdic acid (10 min), and aniline blue (5 min), followed by 1% acetic acid differentiation. Collagen deposition was analyzed using an optical microscope (Nikon, Japan).

### 2.17 Immunofluorescence Staining

Following deparaffinization and rehydration, antigen retrieval was performed using Tris‐EDTA buffer (pH 9.0). The sections were permeabilized with 0.5% Triton X‐100 (15 min, RT) and blocked with 5% donkey serum albumin (1 h, RT). Primary antibody incubation was carried out overnight at 4°C using anti‐COL16A1 (ab231044, abcam; 1:250), anti‐BRG1 (66561‐1‐Ig, Proteintech; 1:250) and anti‐Vimentin (ab8069, abcam; 1:250). Sections were then incubated with Alexa Fluor 488‐conjugated goat anti‐rabbit and Alexa Fluor 555‐conjugated goat anti‐mouse secondary antibodies (Invitrogen; 1:500) for 1 h at RT. The nuclei were counterstained with DAPI. Fluorescence images were acquired using a Nikon Eclipse fluorescence microscope. Immunofluorescence co‐localization analysis was conducted by ImageJ software.

### 2.18 DNA Pull Down plus LC‐MS/MS

Nuclear proteins were extracted from primary mouse dermal fibroblasts cultured in LG medium. Single‐stranded biotinylated probes spanning the COL16A1 promoter‐binding region were synthesized, and nuclear extracts were incubated with probe‐bound Dynabeads C1 Streptavidin (Thermo) overnight at 4°C in binding buffer. The beads were washed, and bound proteins were eluted. The captured proteins were digested with trypsin, desalted, and subjected to liquid chromatography‐tandem mass spectrometry (LC‐MS/MS). Finally, the LC‐MS/MS original data were searched and qualitatively analyzed by using MaxQuant (Version 1.5.6.0). Identifications of ≥2 unique peptides and false discovery rate (FDR) < 1% were required. All proteins meeting these criteria were considered as potential COL16A1 promoter interactors.

### 2.19 Chromatin Immunoprecipitation (ChIP) Assay

ChIP assay was performed using the SimpleChIP Plus Enzymatic Chromatin IP Kit (#9005, CST) with optimized procedures for primary murine fibroblasts. Briefly, cells were washed with PBS and lysed in buffer containing protease inhibitors, omitting formaldehyde crosslinking. Nuclei were pelleted by centrifugation, and chromatin was digested with Micrococcal nuclease to generate DNA fragments (∼150–900 bp). Chromatin samples were immunoprecipitated overnight at 4°C with anti‐BRG1 antibody (66561‐1‐Ig, Proteintech) and normal rabbit IgG as a negative control. The magnetic bead‐bound complexes were washed, DNA was eluted, and crosslink reversal was performed. Purified DNA was analyzed by qPCR using COL16A1‐specific primers (Forward: 5'‐TGGTGCTCTATCCCCTCCTC‐3' and Reverse: 5'‐CTCCATAGTCGTGGAACCCG‐3'). Quantification was performed using standard curves generated from serially diluted input DNA.

### 2.20 Luciferase Reporter Assay

BRG1 overexpression plasmids were constructed using the pcDNA3.1 vector. A luciferase reporter containing the COL16A1 promoter (–2000 kb to +50 kb) was generated by subcloning PCR products into pGL3‐Basic (Genomeditech, Shanghai, China). For the reporter assay, 293T cells were plated in 24‐well plates 1 day before transfection. Following the instructions of the manufacturer, cells were transfected with the overexpression, reporter, and pRL‐TK plasmid. After transfection, cells were lysed, and firefly/Renilla luciferase activities were measured using the Dual‐Luciferase Reporter System (Genomeditech). Promoter activity was expressed as the firefly/Renilla luminescence ratio.

### 2.21 Statistical Analyses

Statistical analyses were performed using GraphPad Prism software (version 8.0; GraphPad Software, USA). Quantitative data from at least three independent experiments are expressed as mean ± standard deviation (SD). Unpaired Student's t‐test were performed for two group comparisons. Following the confirmation of normality and equal variance, one‐way ANOVA with Tukey's post‐hoc test or two‐way ANOVA with Šídák's multiple comparisons test were employed for multiple group comparisons where applicable, with statistical significance set at *p* < 0.05.

## 3 Results

### 3.1 COL16A1 Emerges as a Key Regulator of Fibroblast Dysfunction in DFU from Transcriptomic Analysis

**FIGURE 1:**
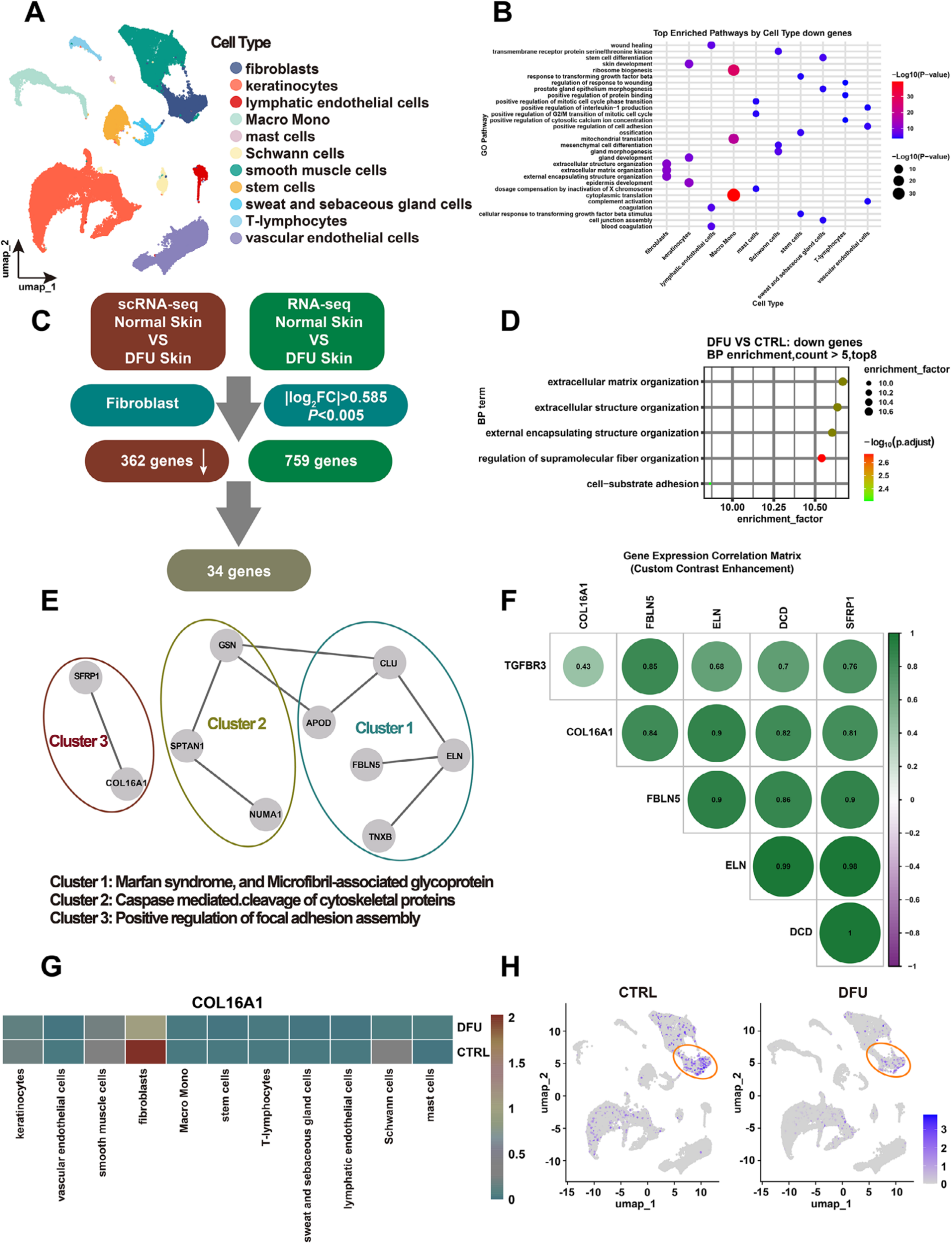
COL16A1 emerges as a key regulator of fibroblast dysfunction in DFU from transcriptomic analysis. (A) Uniform Manifold Approximation and Projection (UMAP) embedding of the entire dataset consisting of 53,234 cells. (B) Cell‐type‐specific enrichment of biological processes for down‐regulated genes (log_2_FC<‐1.5, *p* < 0.05). DEGs in smooth muscle cells did not meet this criteria. For each enriched term, the dot color and size represent the statistical significance, quantified as the ‐log_10_ transformed *p*‐value. (C) Schematic overview of the integrating single‐cell and bulk RNA‐seq datasets analysis. (D) GO enrichment analysis of biological processes for downregulated genes derived from the integrating single‐cell and bulk RNA‐seq datasets. Pathways with fewer than 5 genes were excluded. Results are ordered by enrichment factor, and the significance of P‐values is represented by a color gradient (green to red, reflecting the scale of negative logarithm transformation). (E) PPI network of genes derived from the top 3 enriched pathways, constructed using STRING database. Functional clusters were identified via k‐means algorithm and annotated with representative biological roles. (F) Pairwise Pearson correlation coefficients were calculated for selected genes from three functional modules: extracellular matrix (COL16A1, ELN, FBLN5), signal regulation (TGFBR3, SFRP1), and immune‐matrix interface (DCD). The analysis included all cells from both DFU and CTRL samples. The color gradient represents the correlation coefficient, with deeper green indicating stronger positive correlations and deeper purple indicating stronger negative correlations. (G) Heatmap plots depicting the average expression of COL16A1 across cell types, stratified by clinical groups (DFU and CTRL). Expression levels are visualized via a color gradient (blue: low; red: high), scaled to log_10_(normalized counts + 1). (H) UMAP visualization of COL16A1 expression across all cells. Cells are stratified by clinical groups (DFU and CTRL). Expression intensity is represented by a purple color gradient (light purple: low; dark purple: high), scaled to log_10_(normalized counts + 1).

Recent advances have highlighted the dermal fibroblast's evolving role in diabetic wound healing [28, 29, 30]. To systematically characterize fibroblastic alterations in diabetic wounds, skin specimens from lesional sites of four patients with DFU and healthy skin from four non‐diabetic controls underwent multi‐modal genomic profiling, including single‐cell RNA sequencing (scRNA‐seq) complemented by bulk transcriptomic analysis (the results of Principal Component Analysis (PCA) were supplemented in Figure S1A–C). By integrating high‐resolution single‐cell profiling, our analysis encompassed 53234 cells with rigorously filtered transcriptomes (mitochondrial gene content <20%). Through standardized preprocessing pipelines, we constructed cell‐by‐gene matrices followed by dimension reduction via Uniform Manifold Approximation and Projection and Leiden graph‐based clustering. This multistep computational framework resolved 11 transcriptionally distinct cell populations (Figure 1A; Figure S1D,E). Gene Ontology (GO) Biological Process (BP) enrichment analysis was performed on both up‐ and down‐regulated gene sets for each cell type. Notably, the down‐regulated genes in fibroblasts were significantly enriched in extracellular matrix (ECM)‐related pathways (Figure 1B; Figure S1F). To delineate fibroblast‐specific transcriptional perturbations in DFU microenvironments, we implemented a dual‐omics approach integrating single‐cell and bulk RNA‐seq datasets, with the analytical workflow schematized in Figure 1C. Initial differential expression analysis under stringent thresholds (|log_2_FC| > 0.585, *p* < 0.005) revealed 362 and 759 downregulated genes in respective modalities. Through Venn intersection analysis, we identified 34 consensus DEGs that demonstrated concordant suppression across both transcriptional resolutions. The expression profile of these genes was shown as a clustered heatmap (Figure S1G), underscoring their collective importance in DFU. Notably, these overlapping DEGs exhibited dual regulatory failure as they were transcriptionally compromised within fibroblast subpopulations and exhibited tissue‐wide downregulation in bulk‐level analysis, suggesting their pivotal role in wound repair pathophysiology.

To mechanistically contextualize these findings, GO enrichment analysis of the 34‐gene signature revealed predominant involvement in ECM organization (GO:0030198, FDR = 0.0027) (Figure 1D). Protein‐protein interaction (PPI) network modeling using STRING DB (confidence score > 0.4) further identified three gene clusters within the network (k‐means, n‐cluster = 3) (Figure 1E). Cluster 1 comprised 5 genes associated with microfibril‐associated glycoprotein. Cluster 2 contained genes encoding proteins involved in caspase‐mediated cleavage of cytoskeletal proteins. Notably, genes in cluster 3 (COL16A1 and SFRP1) regulate focal adhesion assembly. This process underpins the fibroblast's core biological functions, directly affecting their survival, migration, contraction, signaling pathways, and responsiveness to the tissue microenvironment [31, 32, 33]. Then we investigated the pairwise correlations between several key genes representing different functional modules: extracellular matrix (COL16A1, ELN, FBLN5), signal regulation (TGFBR3, SFRP1), and the immune‐matrix interface (DCD) (Figure 1F). This robust co‐expression pattern strongly demonstrated that COL16A1 was a vital gene involved in the DFU process. Furthermore, single‐cell transcriptomic analysis revealed the cellular hierarchy of COL16A1 expression, with fibroblasts exhibiting significantly higher expression levels than smooth muscle cells and keratinocytes (Figure 1G). This cell‐type specificity was further pronounced in DFU microenvironments, where fibroblasts exhibited a marked reduction in COL16A1 expression relative to healthy controls (Figure 1H), prompting functional investigations. Combined with the perious research that COL16A1 (FACIT collagen family) participated in stabilizing ECM [18], we assumed that COL16A1 may participate in the wound healing process. This multi‐layered computational evidence, spanning single‐cell resolution, tissue‐level validation, functional annotation, and network topology, established COL16A1 as the prioritized candidate for subsequent mechanical investigation of DFU pathogenesis.

### 3.2 The Expression of Fibroblast‐Derived COL16A1 is Impaired in DFU Wound

**FIGURE 2:**
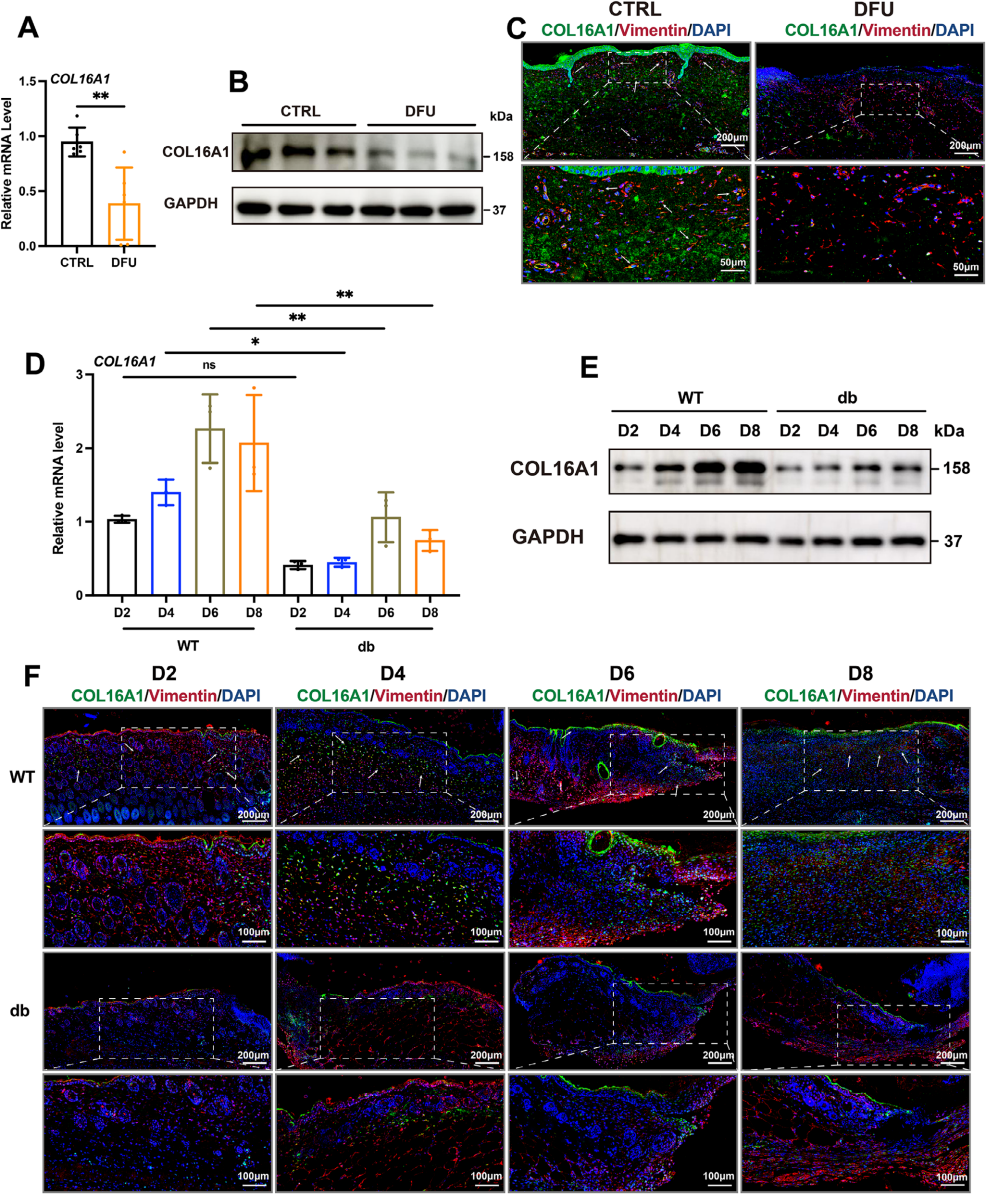
The expression of fibroblast‐derived COL16A1 is impaired in DFU wound. (A, B) Relative mRNA and protein expression level of COL16A1 in CTRL and DFU skin specimens (n = 6). (C) Representative immunofluorescent images of COL16A1 (green) and Vimentin (red) in CTRL and DFU skin specimens. White arrows represent co‐localized area of COL16A1 and Vimentin. Scale bar: 200 and 50 µm. (D, E) Relative mRNA and protein expression level of COL16A1 in wound edge of WT and db/db mice PWD 0 to 8 (n = 3). (F) Representative immunofluorescent images of COL16A1 (green) and Vimentin (red) in wound edge of WT and db/db mice PWD 2 to 8. White arrows represent co‐localized area of COL16A1 and Vimentin. Scale bar: 200 µm. ns: non significance; * *p* < 0.05, ** *p* < 0.01. Data are expressed as mean ± SD. *p*‐values were calculated by the two‐tailed Student's t‐test or one‐way ANOVA test.

Consistent with the transcriptomic results, COL16A1 mRNA expression was significantly decreased in human DFU samples (Figure 2A), a trend corroborated at the protein level (Figure 2B; Figure S2A). Comprehensive correlation analyses demonstrated a statistically significant negative correlation between COL16A1 protein expression and both fasting plasma glucose (FPG) and glycosylated hemoglobin (HbA_1c_) levels in our patient cohort (Figure S2B,C), indicating its potential role as both a biomarker and therapeutic target in DFU progression. Spatial mapping via immunofluorescence demonstrated COL16A1 strong co‐localization with Vimentin (the classic fibroblast cell marker) in healthy tissues, contrasting sharply with DFU tissues, where fibroblast‐specific expression was markedly diminished (Figure 2C; Figure S2D). H&E and Masson staining of human skin specimens also revealed that the lesion in DFU exhibited pronounced inflammatory infiltration, epithelial denudation, and ECM disruption (Figure S2E,F). In murine models, temporal analysis of wound healing revealed divergent expression dynamics as wild‐type (WT) mice exhibited progressive COL16A1 induction during healing, whereas diabetic models exhibited blunted upregulation (Figure 2D,E; Figure S3D), aligning with their delayed wound closure (Figure S3A–C,E,F). Fibroblast‐specific impairment was further supported by reduced dual‐positive cell populations (COL16A1^+^/Vimentin^+^) in diabetic wounds, with expression levels strongly correlating with wound closure rates (Figure 2F; Figure S3G).

Consistent findings from single‐cell resolution to dynamic wound analysis have established COL16A1 dysregulation as a key mechanism in diabetic healing impairment.

### 3.3 COL16A1 Plays a Vital Role in Maintaining Fibroblast Homeostasis and Functional Integrity

**FIGURE 3:**
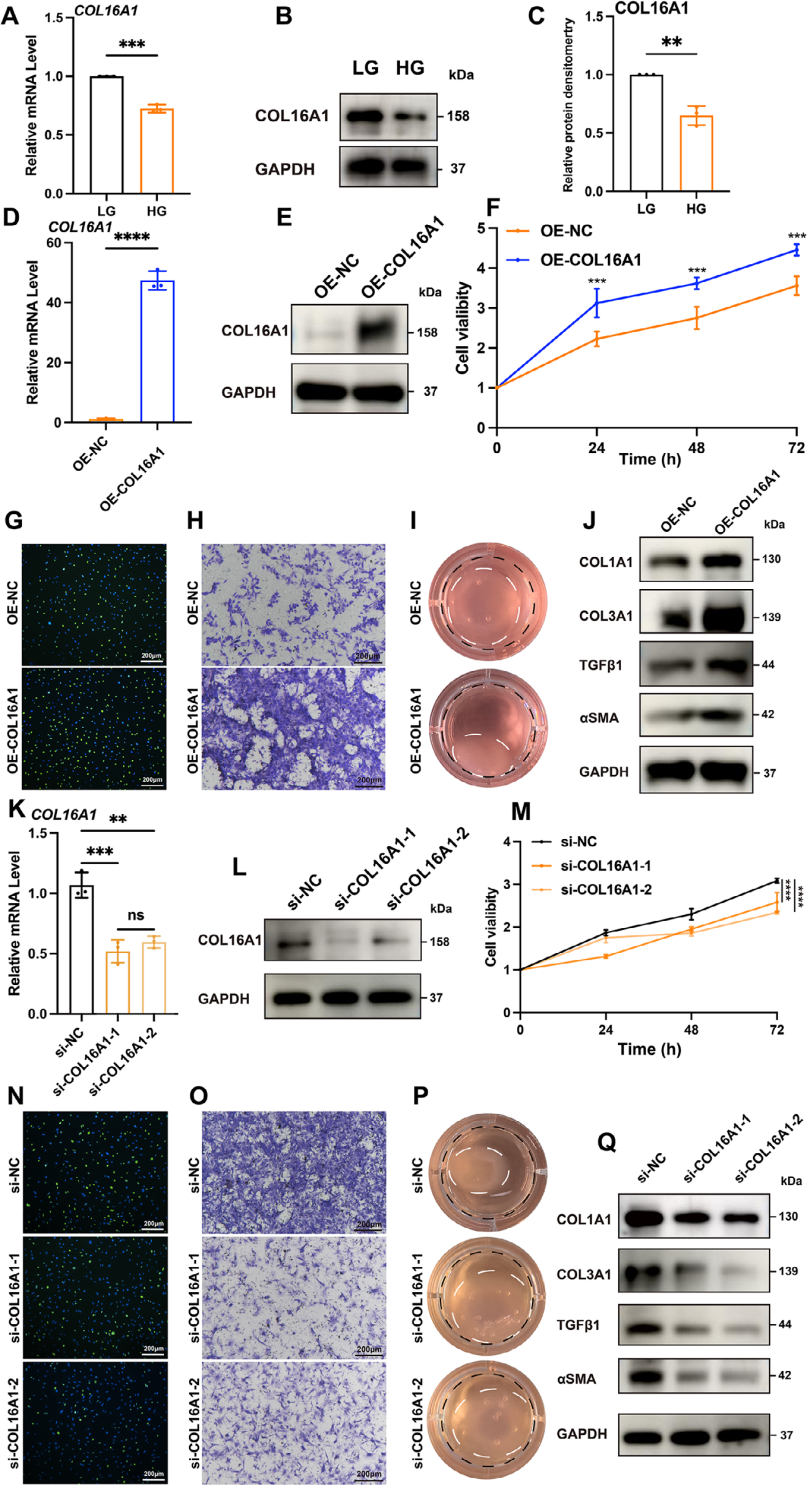
COL16A1 plays a vital role in maintaining fibroblast homeostasis and functional integrity. (A, B) Relative mRNA and protein expression level of COL16A1 in fibroblasts cultured in LG or HG medium (n = 3). (C) Quantification analysis of (B) (n = 3). (D, E) Relative mRNA and protein expression level of COL16A1 in fibroblasts transfected with Ad‐NC and Ad‐OE‐COL16A1 in HG medium (n = 3). (F) CCK‐8 results, (G) EdU (green) proliferation assay (scale bar: 200 µm), (H) Transwell migration assay (scale bar: 200 µm), (I) Cell contraction assay of fibroblasts with COL16A1 overexpression in HG medium. Black circle: area of gels at 0 h; white circle: area of gels at 4 h. (n = 3). (J) Western blot of COL1A1, COL3A1, TGFβ1 and αSMA of fibroblasts with COL16A1 overexpression in HG medium (n = 3). (K, L) Relative mRNA and protein expression level of COL16A1 in fibroblasts transfected with si‐NC and si‐COL16A1 in LG medium (n = 3). (M) CCK‐8 results, (N) EdU (green) proliferation assay (scale bar: 200 µm), (O) Transwell migration assay (scale bar: 200 µm), (P) Cell contraction assay of fibroblasts with COL16A1 knockdown in LG medium. Black circle: area of gels at 0 h; white circle: area of gels at 4 h. (n = 3). (Q) Western blot of COL1A1, COL3A1, TGFβ1 and αSMA of fibroblasts with COL16A1 knockdown in LG medium (n = 3). ns: non significance; ** *p* < 0.01, *** *p* < 0.001, **** *p* < 0.0001. Data are expressed as mean ± SD. *p*‐values were calculated by the two‐tailed Student's t‐test, one‐way or two‐way ANOVA test.

Fibroblasts play critical roles in all phases of wound healing, including inflammatory, proliferative, and remodeling stages, through their functions in migration, proliferation, contraction, and ECM secretion. To validate these observations, we cultured primary mouse dermal fibroblasts under LG (5.5 mmol/L) and HG (25 mmol/L) conditions. Fibroblasts exposed to HG exhibited reduced proliferative capacity (Figure S4A–C), impaired migratory ability (Figure S4D,E), and diminished contractile activity (Figure S4F,G). Concurrently, HG conditions significantly suppressed αSMA and TGFβ1, key marker expression levels associated with fibroblast activation, indicating compromised cellular activation. Furthermore, HG‐cultured fibroblasts demonstrated decreased ECM components synthesis (COL1A1 and COL3A1) (Figure S4H–J). This comprehensive functional inhibition coincided with a significant reduction in COL16A1 expression (Figure 3A–C).

For COL16A1 functional relevance investigation, we modulated its expression in fibroblasts using adenoviral overexpression constructs and siRNA‐mediated knockdown and validated at both mRNA and protein levels (Figure 3D,E,K,L). Under HG conditions, COL16A1 overexpression counteracted glucose‐induced impairments and restored proliferation (Figure 3F,G; Figure S4K), migration (Figure 3H; Figure S4L), contractility (Figure 3I; Figure S4M), and collagen synthesis (Figure 3J; Figure S4N). Conversely, COL16A1 suppression under LG conditions recapitulated the functional deficits observed in HG‐treated cells (Figure 3M–Q; Figure S4O–R). Collectively, these findings demonstrate that COL16A1 is essential for maintaining fibroblast homeostasis and functional integrity.

### 3.4 COL16A1 Accelerates Murine Diabetic Wound Healing

**FIGURE 4:**
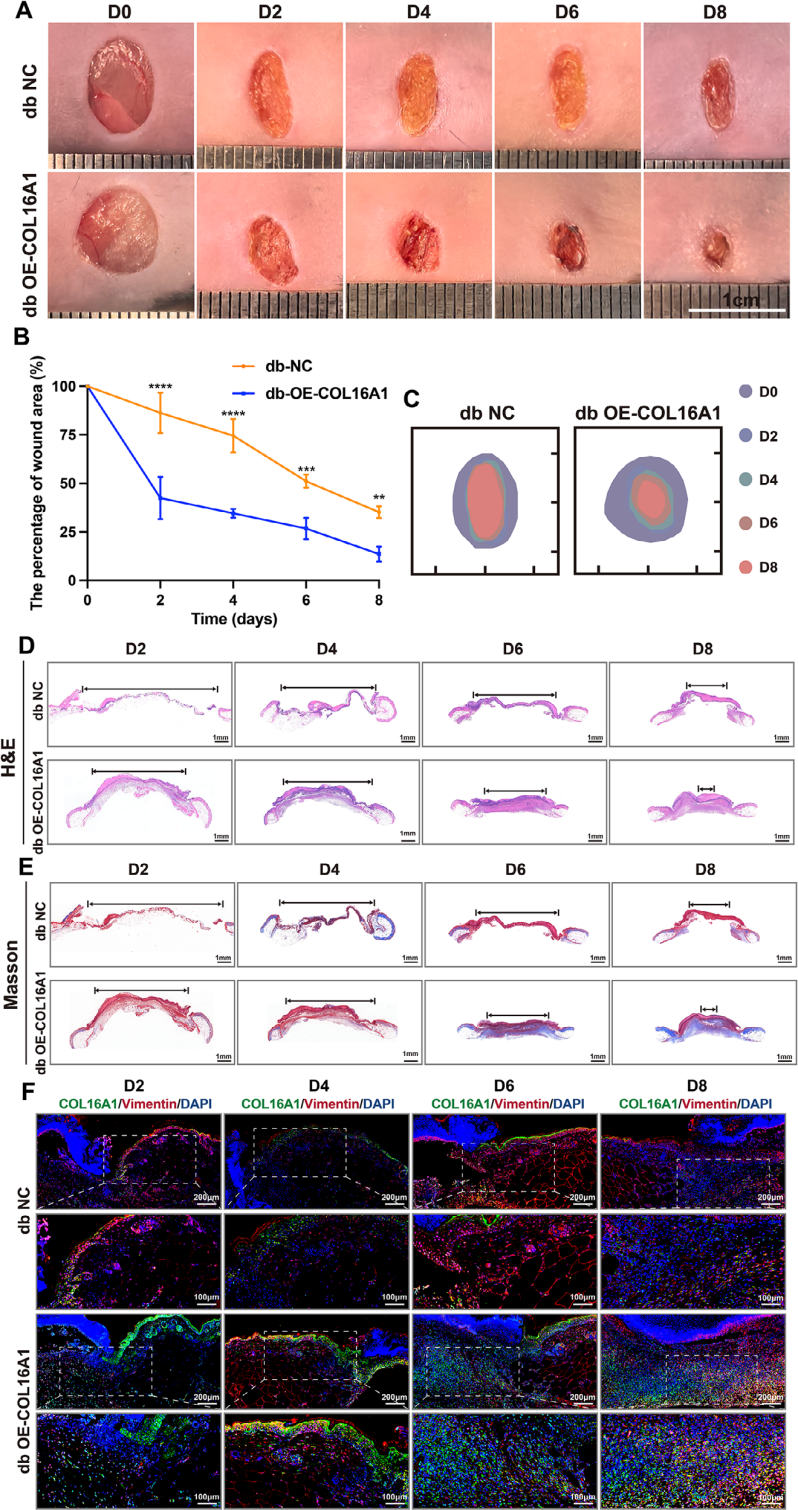
COL16A1 accelerates murine diabetic wound healing. (A, B) Representative chronological images of wounds and analysis of the wound area in Ad‐NC and Ad‐OE‐COL16A1 transfected db/db mice (n = 3). (C) Trace of wound closure PWD 0 to 8. (D, E) Representative images of H&E staining and Masson staining of wounds in Ad‐NC and Ad‐OE‐COL16A1 transfected db/db mice PWD 2 to 8. (F) Representative immunofluorescent images of COL16A1 (green) and Vimentin (red) in Ad‐NC and Ad‐OE‐COL16A1 transfected db/db mice PWD 2 to 8. Scale bar: 200 and 100 µm. ** *p* < 0.01, *** *p* < 0.001, **** *p* < 0.0001. Data are expressed as mean ± SD. *p*‐values were calculated by two‐way ANOVA test.

Given the COL16A1 deficiency in diabetic wounds, we investigated whether COL16A1 topical overexpression could enhance healing in diabetic mice. To test this hypothesis, db/db mice received intradermal adenoviral injections at wound sites to overexpress COL16A1 locally. Compared with control groups, COL16A1‐overexpressing wounds exhibited accelerated closure (Figure 4A)and significantly improved healing rates by PWD 8 (Figure 4B,C). Histological analyses using H&E and Masson staining demonstrated smaller wound beds, near‐complete re‐epithelialization, and enhanced collagen deposition in the COL16A1‐overexpressing group (Figure 4D,E). Until PWD 14, the pro‐healing effects of COL16A1 was sustained with no evidence of wound regression (Figure S5A–E). Immunofluorescence (IF) confirmed increased COL16A1‐positive signals in the dermal layer of treated mice, with co‐localization observed between COL16A1 and the fibroblast marker vimentin (Figure 4F; Figure S5F). These findings demonstrate that localized COL16A1 adenoviral delivery in diabetic skin effectively promoted fibroblast‐specific overexpression, accelerated wound closure, and enhanced re‐epithelialization and collagen deposition.

### 3.5 BRG1 Promotes COL16A1 Transcription and Fibroblast Activation

**FIGURE 5:**
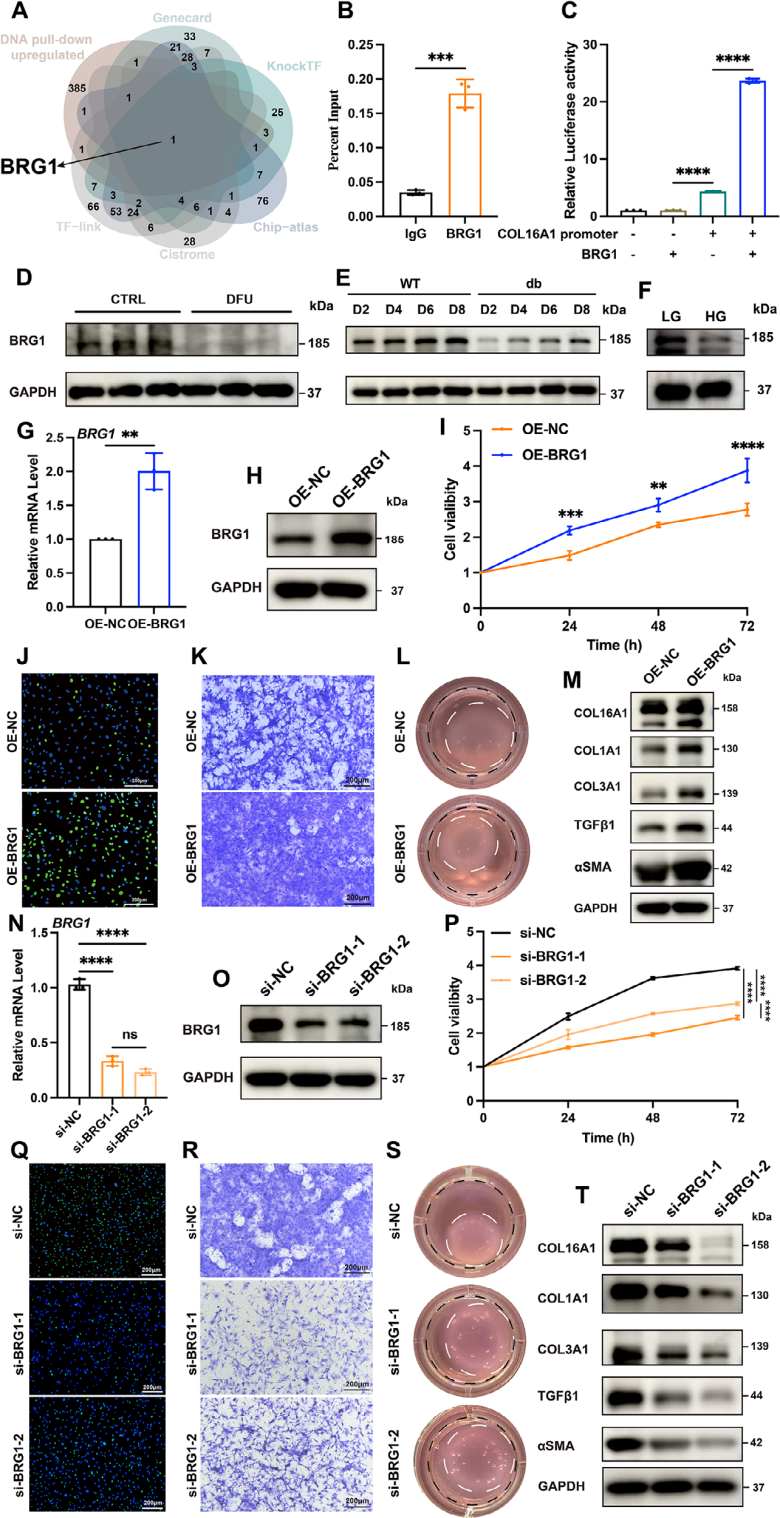
BRG1 promotes COL16A1 transcription and fibroblast activation. (A) Intersection of the DNA pulldown plus LC‒MS/MS upregulated protein assay results with TF‐link, Cistrome, ChIP‐atlas, KnockTF, and GeneCard prediction. (B) ChIP analysis of BRG1 enrichment at the COL16A1 promoter region in fibroblasts cultured with LG medium (n = 3). (C) Luciferase reporter assay of COL16A1 transcriptional activity with or without BRG1 transfection (n = 3). Western blot of BRG1 in (D) CTRL and DFU skin specimens, (E) wound edge of WT and db/db mice PWD 2 to 8, (F) fibroblasts cultured in LG or HG medium. (G, H) Relative mRNA and protein expression level of BRG1 in fibroblasts transfected with Ad‐NC and Ad‐OE‐BRG1 in HG medium (n = 3). (I) CCK‐8 results, (J) EdU (green) proliferation assay (scale bar: 200 µm), (K) Transwell migration assay (scale bar: 200 µm), (L) Cell contraction assay of fibroblasts with BRG1 overexpression in HG medium. Black circle: area of gels at 0 h; white circle: area of gels at 4 h. (n = 3). (M) Western blot of COL16A1, COL1A1, COL3A1, TGFβ1 and αSMA of fibroblasts with BRG1 overexpression in HG medium (n = 3). (N, O) Relative mRNA and protein expression level of BRG1 in fibroblasts transfected with si‐NC and si‐BRG1 in LG medium (n = 3). (P) CCK‐8 results, (Q) EdU (green) proliferation assay (scale bar: 200 µm), (R) Transwell migration assay (scale bar: 200 µm), (S) Cell contraction assay of fibroblasts with BRG1 knockdown in LG medium. Black circle: area of gels at 0 h; white circle: area of gels at 4 h. (n = 3). (T) Western blot of COL16A1, COL1A1, COL3A1, TGFβ1 and αSMA of fibroblasts with BRG1 knockdown in LG medium (n = 3). ns: non significance; ** *p* < 0.01, *** *p* < 0.001, **** *p* < 0.0001. Data are expressed as mean ± SD. *p*‐values were calculated by the two‐tailed Student's t‐test, one‐way or two‐way ANOVA test.

Given the COL16A1's important role in wound repair, we explored its upstream regulatory mechanisms to identify potential therapeutic targets for chronic diabetic wounds. Initial high‐throughput screening identified transcription factors (TFs) binding to the COL16A1 promoter. Using DNA probes targeting the COL16A1 promoter region, protein pulldown assays in primary mouse dermal fibroblasts cultured under LG conditions revealed 385 candidate proteins through LC‐MS/MS. Cross‐referencing with predictions from five public TF databases (TF‐link, Cistrome, ChIP‐atlas, KnockTF, and GeneCard), we prioritized BRG1 (SMARCA4) as the most plausible COL16A1 regulator (Figure 5A).

For direct binding validation, ChIP‐qPCR in LG‐cultured fibroblasts demonstrated significant BRG1 enrichment at predicted binding sites within the COL16A1 promoter than IgG controls (Figure 5B). To assess transcriptional activation, we cloned the COL16A1 promoter (−2000 to +50 bp) into a pGL3‐luciferase reporter vector. BRG1 overexpression markedly increased luciferase activity, confirming its role in transactivating COL16A1 expression (Figure 5C). To precisely map the BRG1 binding sites on the COL16A1 promoter, we mutated two predicted sites at positions −751 to −758 bp and −258 to −265 bp upstream of the transcription start site, and conducted luciferase assays (Figure S6A,B), demonstrating that mutation of either site significantly reduced promoter activity compared to the wild‐type control. Collectively, these findings establish BRG1 as a COL16A1 direct transcriptional activator through promoter binding.

To elucidate the BRG1 mechanism in diabetic wound healing, we first analyzed its expression patterns in clinical specimens. Western blot revealed BRG1 protein levels significant downregulation in DFU tissues compared to normal tissues (Figure 5D; Figure S6C). Consistent with human data, diabetic murine models exhibited reduced BRG1 expression at wound margins relative to normal murine models (Figure 5E; Figure S6D). In vitro analyses further demonstrated suppressed BRG1 expression in fibroblasts cultured under HG conditions (Figure 5F; Figure S6E). These observations led us to hypothesize that hyperglycemia‐induced BRG1 deficiency disrupts COL16A1 transcriptional activation, thereby impairing wound repair processes.

For this hypothesis investigation, we engineered adenoviral constructs for BRG1 overexpression and siRNA‐mediated knockdown, with transfection efficiency validated through RT‐qPCR and western blot in primary fibroblasts (Figure 5G,H,N,O; Figure S6F,O). Under HG conditions, BRG1 overexpression significantly restored fibroblast proliferative capacity (Figure 5I,J; Figure S6G), enhanced migration (Figure 5K; Figure S6H), and improved contractile function (Figure 5L; Figure S6I). Concurrently, BRG1 overexpression upregulated COL16A1 expression (Figure 5M; Figure S6J) and promoted COL1A1 and COL3A1 synthesis (Figure 5M; Figure S6K–N).

Conversely, BRG1 knockdown in LG environments inhibited COL16A1 expression (Figure 5T; Figure S6S) and recapitulated the HG‐induced functional deficits, including impaired proliferation (Figure 5P,Q; Figure S6P), migration (Figure 5R; Figure S6Q), contractile function (Figure 5S; Figure S6R), and collagen production (Figure 5T; Figure S6T–W). These data collectively establish that BRG1 serves as COL16A1's critical transcriptional regulator, orchestrating fibroblast‐mediated ECM remodeling essential for diabetic wound resolution.

### 3.6 BRG1 Restores Fibroblast‐Mediated COL16A1 Secretion and Promotes Murine Diabetic Wound Healing

**FIGURE 6:**
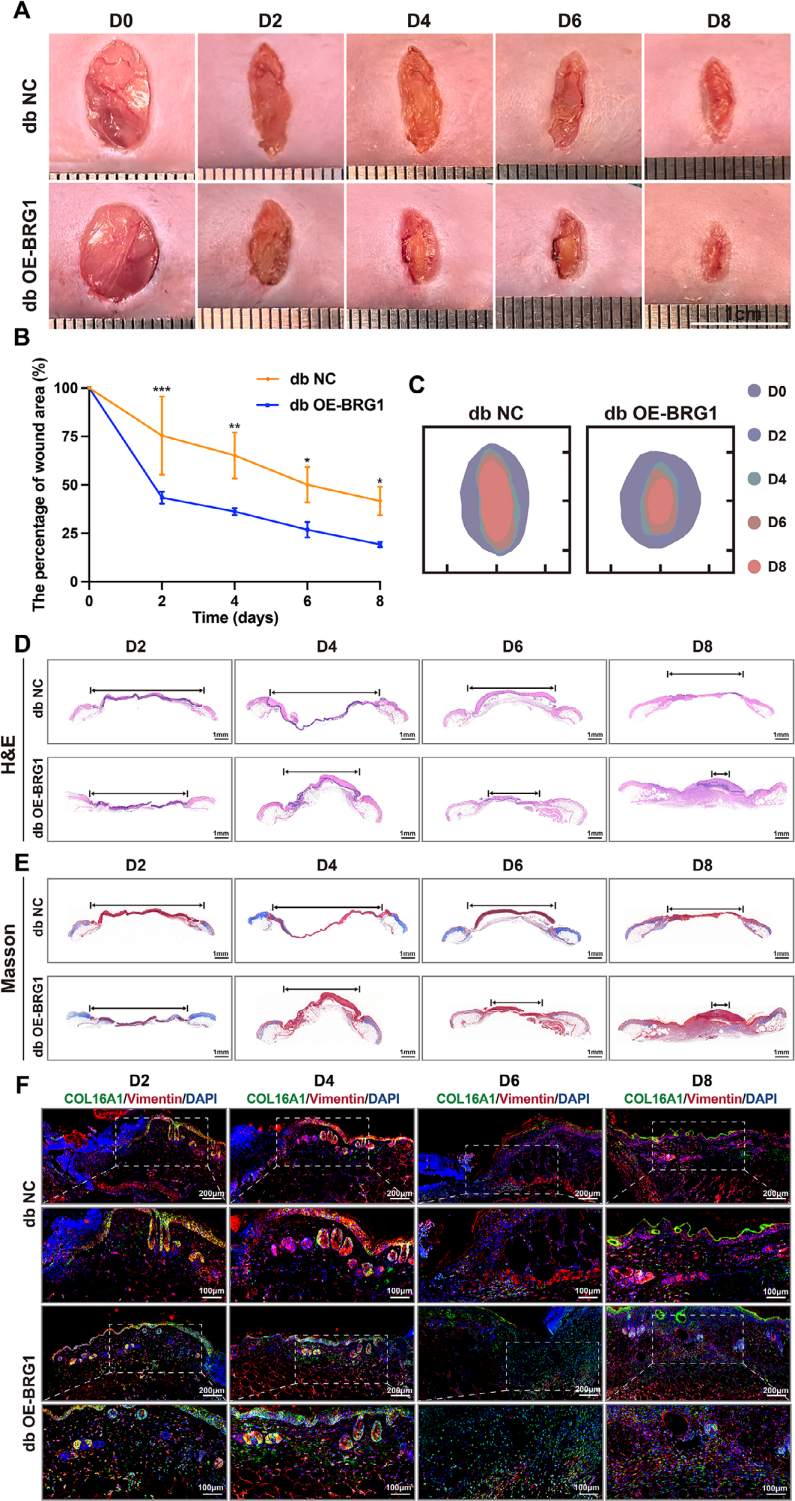
BRG1 restores fibroblast‐mediated COL16A1 secretion and promotes murine diabetic wound healing. (A, B) Representative chronological images of wounds and analysis of the wound area in Ad‐NC and Ad‐OE‐BRG1 transfected db/db mice (n = 3). (C) Trace of wound closure PWD 0 to 8. (D, E) Representative images of H&E staining and Masson staining of wounds in Ad‐NC and Ad‐OE‐BRG1 transfected db/db mice PWD 2 to 8. (F) Representative immunofluorescent images of COL16A1 (green) and Vimentin (red) in Ad‐NC and Ad‐OE‐BRG1 transfected db/db mice PWD 2 to 8. Scale bar: 200 and 100 µm. ns: non significance, * *p* < 0.05, ** *p* < 0.01, *** *p* < 0.001. Data are expressed as mean ± SD. *p*‐values were calculated by two‐way ANOVA test.

For BRG1 therapeutic potential investigation in diabetic wound healing, we performed localized adenoviral‐mediated BRG1 overexpression in the diabetic mice's dorsal skin. IF analysis confirmed enhanced BRG1^+^ signals at wound margins than negative control groups, validating successful BRG1 reconstitution in vivo (Figure S7A). Longitudinal wound measurements demonstrated accelerated closure rates in BRG1‐overexpressing diabetic mice than untreated diabetic counterparts (Figure 6A–C; Figure S7B–D). Histopathological evaluation via H&E and Masson staining revealed pronounced wound bed contraction, accelerated re‐epithelialization, and augmented collagen deposition in the BRG1 overexpression group (Figure 6D,E; Figure S7E,F). Based on in vitro evidence of BRG1 binding to the COL16A1 promoter, we further assessed COL16A1 expression in vivo. Analysis exhibited marked COL16A1 protein upregulation at wound margins post‐BRG1 overexpression, with partial co‐localization observed between COL16A1 and the fibroblast marker Vimentin (Figure 6F; Figure S7G). These findings demonstrated that localized BRG1 overexpression in diabetic skin restored fibroblast‐mediated COL16A1 secretion, promoted wound bed contraction, and enhanced healing kinetics. These results indicated that BRG1‐driven COL16A1 transactivation leads to functional improvements in diabetic wound repair.

### 3.7 The Pro‐Fibrotic Effects of BRG1 Depends on COL16A1

**FIGURE 7:**
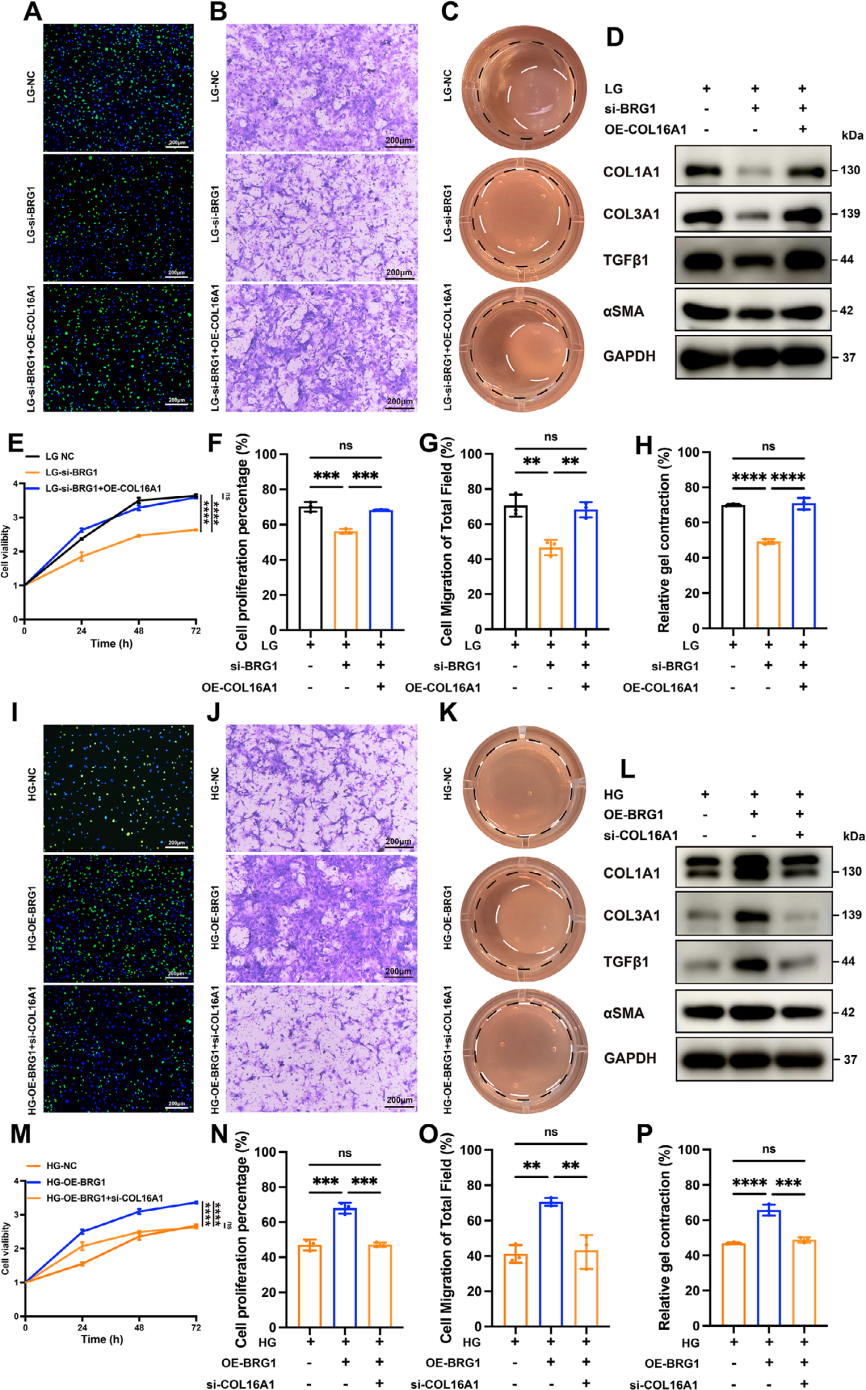
The pro‐fibrotic effects of BRG1 depends on COL16A1. (A) EdU (green) proliferation assay (scale bar: 200 µm), (B) Transwell migration assay (scale bar: 200 µm), (C) Cell contraction assay. Black circle: area of gels at 0 h; white circle: area of gels at 4 h, (D) Western blot of COL1A1, COL3A1, TGFβ1 and αSMA (E) CCK‐8 results in BRG1 knockdown fibroblasts with COL16A1‐overexpression in LG medium (n = 3). (F–H) Quantification analysis of (A), (B) and (C), respectively (n = 3). (I) EdU (green) proliferation assay (scale bar: 200 µm), (J) Transwell migration assay (scale bar: 200 µm), (K) Cell contraction assay. Black circle: area of gels at 0 h; white circle: area of gels at 4 h, (L) Western blot of COL1A1, COL3A1, TGFβ1 and αSMA (M) CCK‐8 results in BRG1‐overexpression fibroblasts with COL16A1 knockdown in HG medium (n = 3). (N–P) Quantification analysis of (I), (J) and (K), respectively (n = 3). ns: non significance; * *p* < 0.05, ** *p* < 0.01, *** *p* < 0.001, **** *p* < 0.0001. Data are expressed as mean ± SD. *p*‐values were calculated by the two‐tailed Student's t‐test, one‐way or two‐way ANOVA test.

To further confirm the BRG1 pro‐fibrotic effects dependency on COL16A1, we conducted complementary in vitro rescue experiments. Primary fibroblasts subjected to BRG1 knockdown under LG conditions exhibited impaired biological functions (proliferation, migration, and contractility), consistent with previous findings. Subsequent COL16A1 overexpression in BRG1 knockdown cells significantly rescued functional deficits and restored collagen synthesis (COL1A1/COL3A1) (Figure 7A–H; Figure S8A), suggesting COL16A1 exogenous overexpression counteracted the antifibrotic effects of BRG1 knockdown in vitro. Conversely, under HG conditions, BRG1‐overexpressing fibroblasts with COL16A1 knockdown demonstrate attenuated activation markers, diminished proliferative capacity, impaired migration, and reduced contractile function (Figure 7I–P; Figure S8B), indicating COL16A1 inhibition suppressed the pro‐fibrotic effect of BRG1 in vitro. These results conclusively demonstrated that the BRG1‐COL16A1 axis is critical for regulating fibroblast function.

## 4 Discussion

DFU, as a type of chronic wound, imposes heavy medical and economic burdens on society due to its high disability and mortality rates [34]. In this study, we elucidated the vital regulatory role of fibroblast‐derived COL16A1 in DFU wound healing and identified BRG1 as a COL16A1 expression key mediator in DFU repair. In normal wound healing, the transcription factor BRG1 is upregulated and directly binds to the COL16A1 promoter, enhancing its expression. This activation promotes fibroblast proliferation, migration, and contraction at wound margins, increases ECM deposition, accelerates granulation tissue formation, and wound closure. However, the BRG1‐COL16A1 axis was markedly impaired in diabetic wounds. Local overexpression of either BRG1 or COL16A1 effectively restored diabetic wound repair.

Previous single‐cell sequencing studies have identified significant pathological alterations in DFU, including compromised re‐epithelialization, dysregulated inflammatory responses, impaired granulation tissue formation, and hindered angiogenesis [29]. Our work demonstrated that COL16A1 may serve as a critical regulator of fibroblast‐mediated DFU healing. Fibroblasts play essential roles in all wound healing phases, encompassing recruiting immune cells through chemokine secretion, activating and migrating to wound beds, secreting ECM components, forming stress fibers for wound contraction, and orchestrating matrix remodeling [35, 36]. Under hyperglycemic conditions, fibroblast activation is suppressed, accompanied by impaired proliferation, migration, contraction, and reduced ECM production [37], findings consistent with our results. Collagen constitutes an essential structural protein in skin, representing 75% of cutaneous dry weight. This protein superfamily, comprising evolutionarily conserved macromolecules, not only exhibits structural diversity but also performs multifunctional roles in ECM [38]. During wound healing, fibroblasts actively synthesize and secrete collagen I and III to mediate tissue defect repair, thereby providing toughness and strength to skin [39]. In the early stages of this process, collagen V and VI production regulate dermal fibroblast migration and proliferation [40, 41]. Meanwhile, collagen IV and VII at the dermal‐epidermal junction stabilize basement membrane structure to facilitate re‐epithelization [42, 43]. Furthermore, collagen XVII — a pivotal hemidesmosome component — promotes keratinocyte migration, proliferation, and adhesion, ultimately coordinating wound re‐epithelialization [44]. As a member of the FACIT collagen family, COL16A1 maintains ECM structural integrity via integrin binding, which facilitates cell‐matrix communication and modulates cell migration, proliferation, and tissue remodeling [45, 46]. While COL16A1 has been implicated in fibrotic diseases and cancer (localized and systemic sclerosis, Epidermolysis bullosa, Crohn's disease, and oral squamous cell carcinoma [17, 47, 48, 49]), its role in diabetic wound healing remained unexplored. By manipulating the COL16A1 expression, we confirmed that COL16A1 overexpression enhanced fibroblast functionality and improved diabetic wound closure, whereas COL16A1 knockdown exacerbated healing deficits. Our findings extended the understanding of COL16A1 in wound healing beyond its canonical role as a FACIT collagen in stabilizing ECM, revealing its capacity to directly activate dermal fibroblasts and thereby promote the healing of diabetic wounds.

For COL16A1 upstream regulatory mechanism investigation, we employed DNA pulldown coupled with LC‐MS/MS, ChIP‐qPCR, and luciferase reporter assays, identifying BRG1 as a COL16A1 direct transcriptional activator. BRG1, the catalytic core of the SWI/SNF chromatin remodeling complex, mechanistically regulates transcriptional programs by generating nucleosome‐depleted regions through ATP‐dependent nucleosome ejection and/or sliding [50, 51, 52]. While BRG1 exhibits context‐dependent transcriptional activation/repression roles [53, 54, 55, 56] and is known to regulate hair follicle stem cell maintenance [25] and keratinocyte migration [24] for tissue regeneration, its involvement in fibroblast‐mediated COL16A1 expression and diabetic wound healing remains unclear. We demonstrated that BRG1 overexpression under HG conditions restored COL16A1 expression, enhanced fibroblast function, and accelerated diabetic wound healing. Conversely, BRG1 knockdown in LG conditions suppressed COL16A1 expression and impaired cellular functionality. Simultaneous BRG1 overexpression/knockdown with COL16A1 knockdown/overexpression revealed that the BRG1 effects on fibroblast behavior and diabetic wound healing were strictly COL16A1‐dependent.

However, our study has the following limitations. First, while we focused on the BRG1‐COL16A1 axis role in wound healing, the precise mechanism by which COL16A1 influences fibroblast function remains unclear. Previous studies have demonstrated that integrin adhesion receptors bind ECM ligands on the cell surface and subsequently assemble adhesion complexes through interactions with intracellular Talin, thereby linking to the cytoskeleton. Enhanced cell‐ECM adhesion regulates cellular functions and promotes wound repair [57]. We hypothesized that COL16A1 may similarly modulate fibroblast activity through analogous mechanisms, although this requires further validation. Second, our in vivo validation was limited to a murine dorsal excisional wound healing model. It is well established that substantial differences exist between murine and human wound healing processes, as mouse skin exhibits greater laxity with a panniculus carnosus layer, predisposing to wound contraction [58], while human skin demonstrates prolonged inflammatory and extended proliferative phases marked by robust granulation tissue formation [59]. Third, our current in vivo approach utilized local intradermal injection of adenovirus‐mediated gene overexpression. While this approach can achieve gene overexpression in local tissues, it lacks cell type specificity. Future studies employing inducible, fibroblast‐specific genetic models or engineer targeted delivery vehicles (for example, homologous membrane‐coated nanoparticles) will be invaluable to achieve cell‐specific modulation of gene regulation in wound‐bed fibroblasts.

In conclusion, our study identified fibroblast‐derived COL16A1 as a critical regulator of DFU healing. We first reported that BRG1 directly activates COL16A1 transcription by binding its promoter region, thereby enhancing fibroblast functionality and diabetic wound repair. These findings provide novel therapeutic targets for chronic diabetic wounds.

## 5 Conclusions

In this study, we identified fibroblast‐derived COL16A1 as a critical mediator of DFU wound healing through analysis of human skin specimens. In vitro experiments demonstrated that COL16A1 activated fibroblasts, enhancing their proliferative, migratory, and contractile capacities while promoting ECM secretion. In vivo studies further confirmed that COL16A1 overexpression accelerated diabetic wound closure. Besides, BRG1 directly binds to the COL16A1 promoter region, driving its transcriptional activation in fibroblasts and thereby facilitating wound repair. These findings elucidate a BRG1‐COL16A1 regulatory axis as a pivotal therapeutic target for impaired diabetic wound healing.

## Author Contributions

P.W. performed conceptualization, methodology, validation, and wrote the original draft. J.S. performed formal analysis, investigation, and wrote the original draft. Y.W. performed software, formal analysis, and visualization. B.R., Y.L., L.C., and L.Y. performed validation and investigation. Y.L. performed validation and data curation. T.D. and L.Y. performed supervision and project administration. Z.D. performed conceptualization, supervision, project administration, funding acquisition, wrote, review and edited the final manuscript.

## Conflicts of Interest

The authors declare no conflicts of interest.

## Supporting information




**Supporting File**: advs74596‐sup‐0001‐SuppMat.docx

## Data Availability

The data that support the findings of this study are available from the corresponding author upon reasonable request.
