# Supplementary material for: Promotion of DFU Wound Healing via BRG1–COL16A1 Axis in Fibroblasts
Source: Adv Sci (Weinh). 2026 Mar 2;13(26):e16687. doi: 10.1002/advs.202516687 (PMC13159164; doi:10.1002/advs.202516687)
Supplement: Supplementary file 1 — Supporting File: advs74596‐sup‐0001‐SuppMat.docx [file ADVS-13-e16687-s001.docx]

**Figure S1 COL16A1 emerges as a key regulator of fibroblast dysfunction in DFU from transcriptomic analysis.**

(A) PCA of bulk RNA-seq. PCA plots visualizing cell distributions before and after data integration: (B) Pre-integration, cells are colored by sample of origin, illustrating the presence of batch effects. (C) Post-integration, patient-specific batches are removed, revealing a more integrated cell distribution. (D) Distribution and expression of representative genes in each cell type, showing by violin plot. (E) Analysis of cell type abundance differences between DFU and CTRL groups. For each cell type, the distribution of its proportion across samples is shown. Individual points correspond to sample-level proportions (CTRL, blue; DFU, yellow). A Wilcoxon rank-sum test was applied to determine the statistical significance of the differences observed between the two groups. (F) Cell-type-specific enrichment of biological processes for up-regulated genes. For each enriched term, the dot color and size represent the statistical significance, quantified as the -log_10_ transformed p-value. (G) Clustered heatmap of the 34 DEGs. Sample-level average expression values were computed with Seurat's AggregateExpression function (v5). To emphasize gene-specific expression patterns between DFU and CTRL conditions, values were standardized per row (z-scoring) prior to plotting.


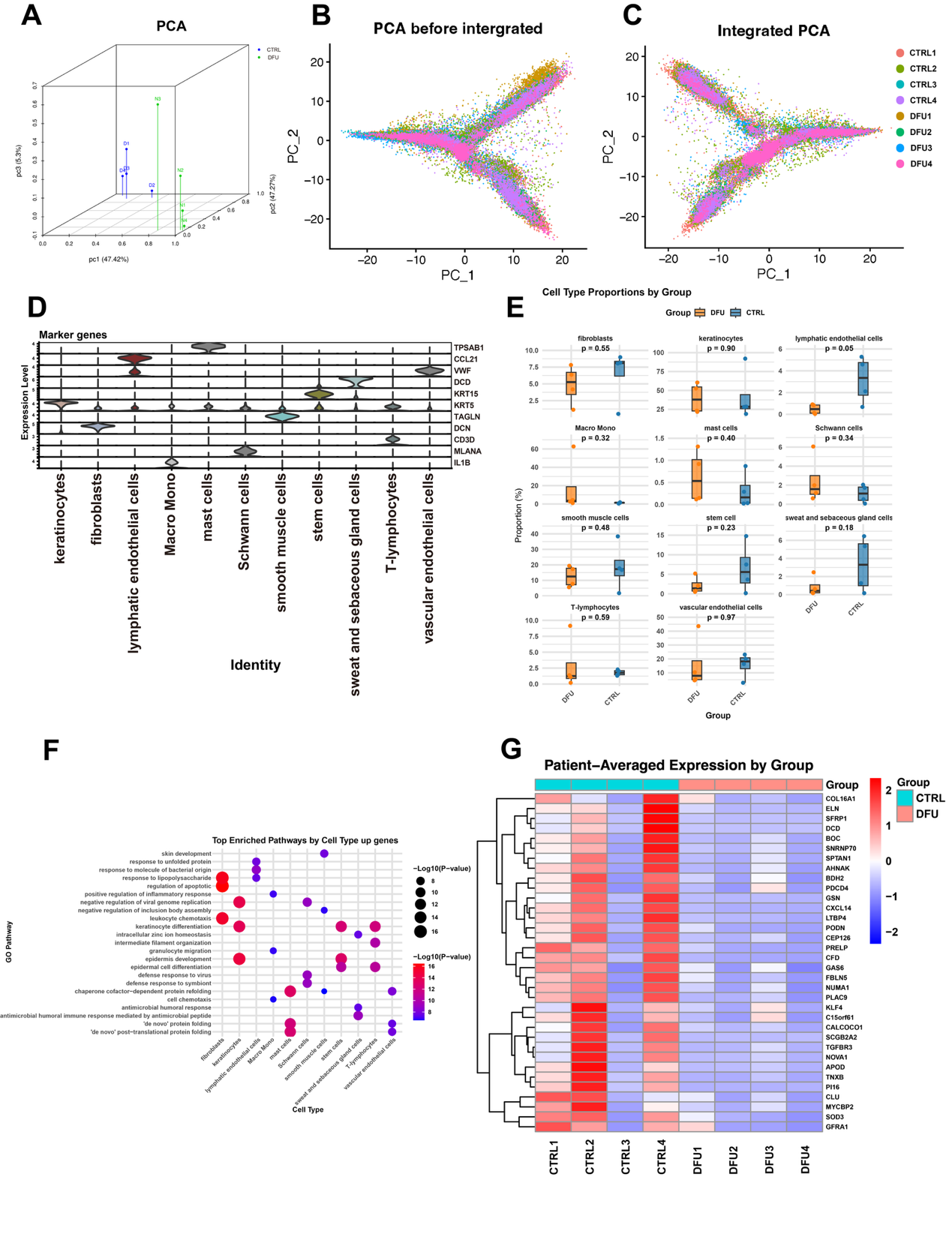


**Figure S2 The expression of fibroblast-derived COL16A1 is impaired in DFU wound.**

(A) Quantification analysis of protein expression level of COL16A1 in CTRL and DFU patients skin specimens (n=6). (B-C) Comprehensive correlation analyses between COL16A1 protein expression and both fasting plasma glucose (FPG) and glycosylated hemoglobin (HbA1c) levels in DFU (n=6) and CTRL (n=6). (D) Immunofluorescence co-localization analysis of COL16A1 (green) and Vimentin (red) in CTRL and DFU skin specimens. (E-F) Representative images of H&E staining and Masson staining of CTRL and DFU skin specimens. **** *p* < 0.0001. Data are expressed as mean ± SD. *p*-values were calculated by the two-tailed Student’s t-test.


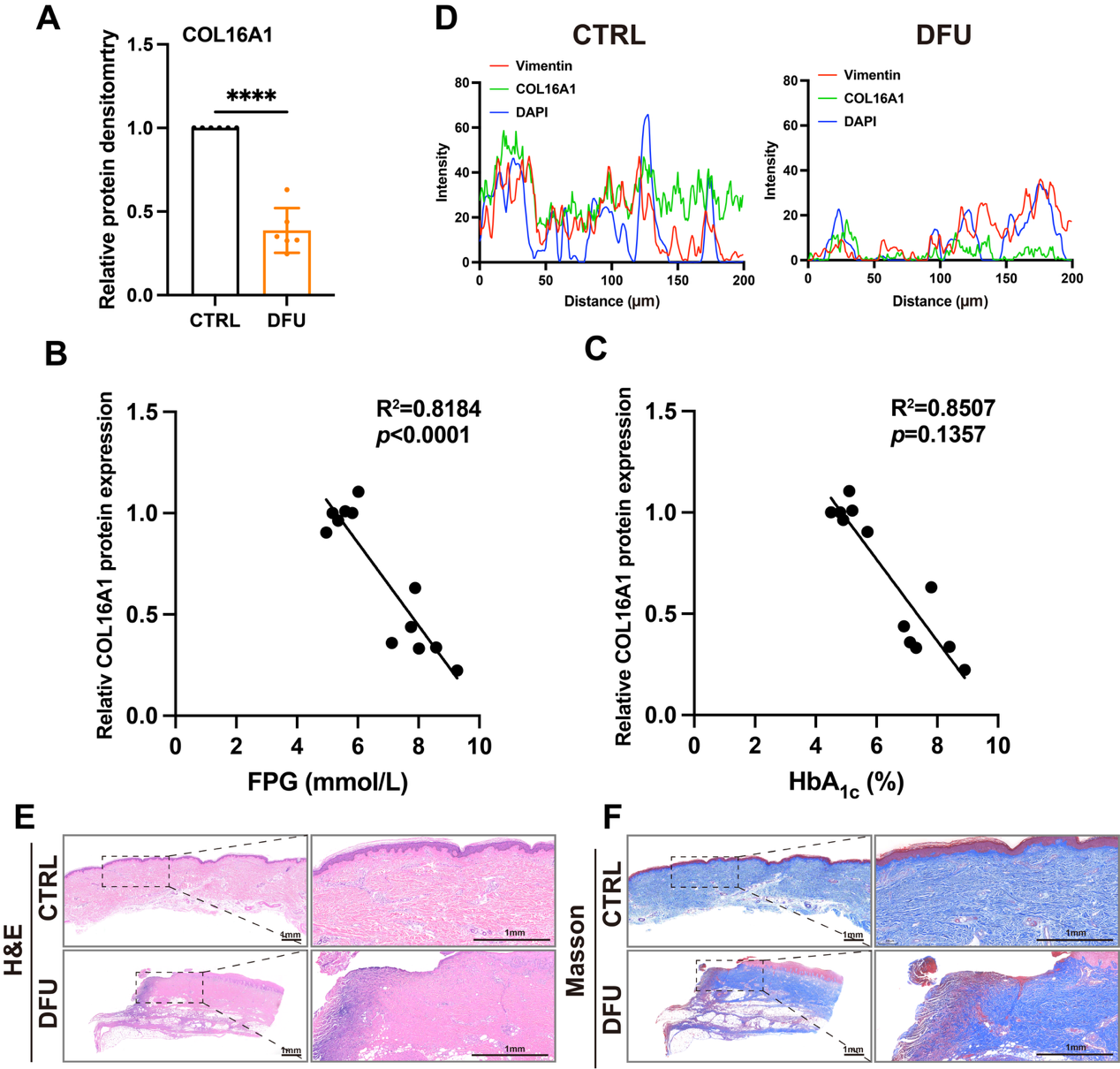


**Figure S3 The expression of fibroblast-derived COL16A1 is impaired in diabetic mice wounds.**

(A-B) Representative chronological images of wounds and analysis of the wound area in WT and db/db mice (n=3). (C) Trace of wound closure PWD 0 to 8. (D) Quantification analysis of protein expression level of COL16A1 in wound edge of WT and db/db mice PWD 2 to 8 (n=3). (E-F) Representative images of H&E staining and Masson staining of wounds in WT and db/db mice PWD 2 to 8. (G) Immunofluorescence co-localization analysis of COL16A1 (green) and Vimentin (red) in WT and db/db mice PWD 2 to 8. ns: non significance; ** *p* < 0.01, *** *p* < 0.001, **** *p* < 0.0001. Data are expressed as mean ± SD. *p*-values were calculated by the two-tailed Student’s t-test or one-way ANOVA test.

**
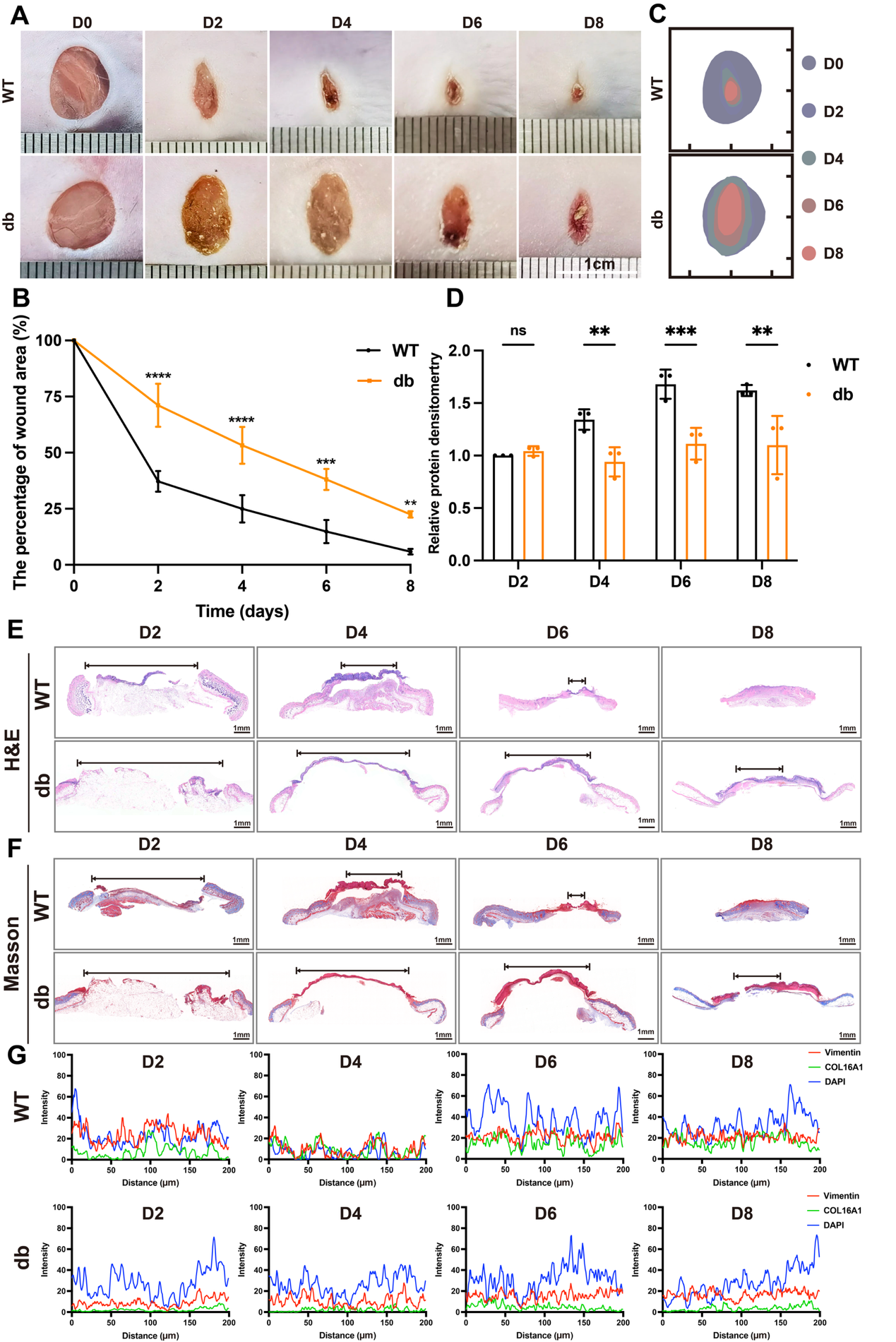
**

**Figure S4 COL16A1 plays a vital role in maintaining fibroblast homeostasis and functional integrity.**

(A) CCK-8 results, (B) EdU (green) proliferation assay (scale bar: 200 μm), (D) Transwell migration assay (scale bar: 200 μm), (F) Cell contraction assay of fibroblasts cultured in LG or HG medium (n=3). (C), (E) and(G) Quantification analysis of (B), (D) and (F), respectively (n=3). (H-I) Relative mRNA and protein expression level of COL16A1 in fibroblasts cultured in LG or HG medium (n=3). (J) Quantification analysis of (I) (n=3). (K-N) Quantification analysis of EdU proliferation assay, Transwell migration assay, cell contraction assay and protein expression level of fibroblasts with COL16A1 overexpression in HG medium (n=3). (O-R) Quantification analysis of EdU proliferation assay, Transwell migration assay, cell contraction assay and protein expression level of fibroblasts with COL16A1 knockdown in LG medium (n=3). ns: non significance; * *p* < 0.05, ** *p* < 0.01, *** *p* < 0.001, **** *p* < 0.0001. Data are expressed as mean ± SD. *p*-values were calculated by the two-tailed Student’s t-test, one-way or two-way ANOVA test.

**
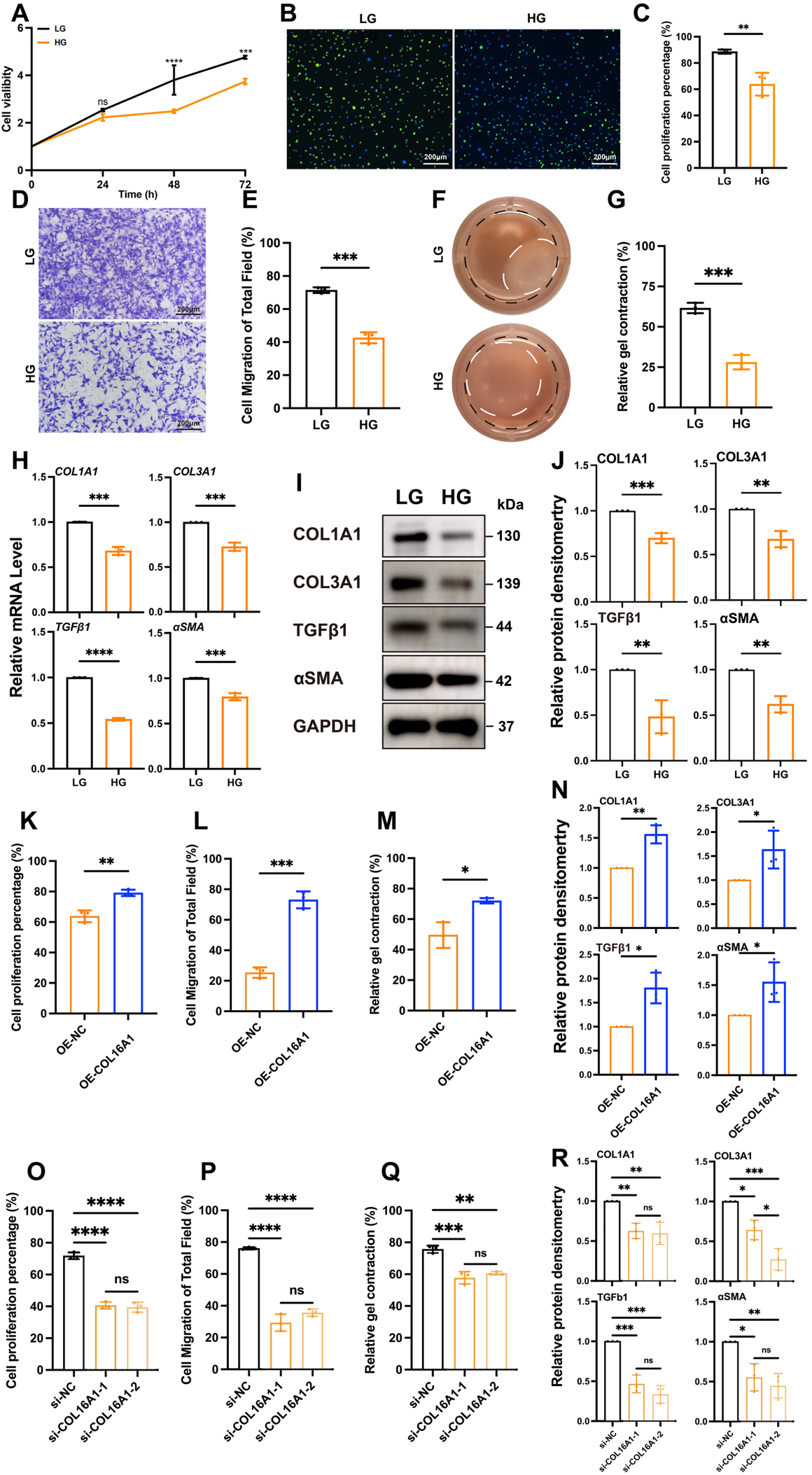
**

**Figure S5 COL16A1 accelerates murine diabetic wound healing.**

(A-B) Representative chronological images of wounds and analysis of the wound area in Ad-NC and Ad-OE-COL16A1 transfected db/db mice (n=3). (C) Trace of wound closure PWD 0 to 14. (D-E) Representative images of H&E staining and Masson staining of wounds in Ad-NC and Ad-OE-COL16A1 transfected db/db mice PWD 4 to 14. (A) Immunofluorescence co-localization analysis of COL16A1 (green) and Vimentin (red) in Ad-NC and Ad-OE-COL16A1 transfected db/db mice. ns: non significance; *** *p* < 0.001, **** *p* < 0.0001. Data are expressed as mean ± SD. *p*-values were calculated by two-way ANOVA test.

**
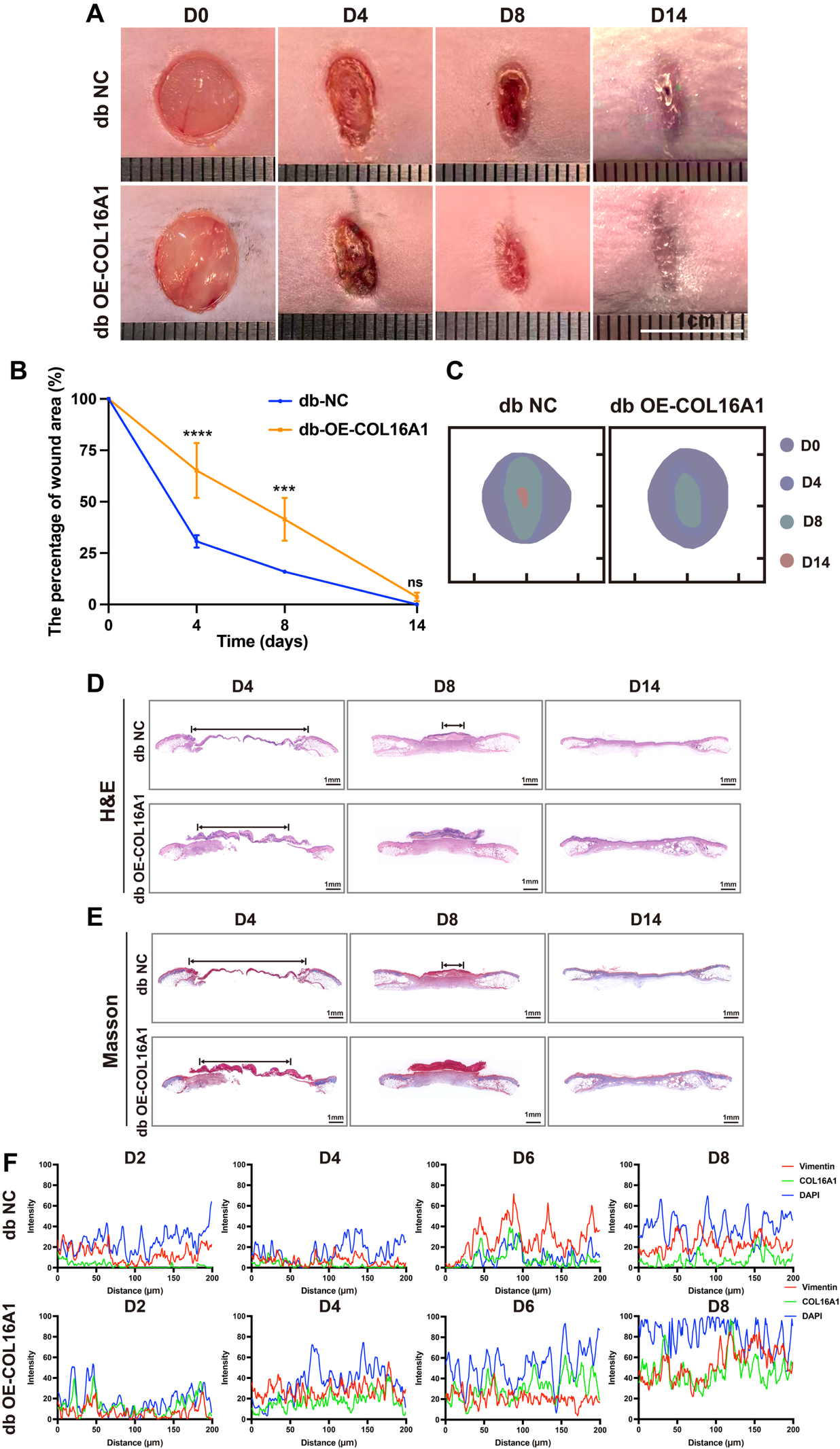
**

**Figure S6 BRG1** **promotes COL16A1 transcription and fibroblast activation.**

(A) Two potential BRG1 binding sites within the COL16A1 promoter region were predicted through JASPAR transcription factor binding profile database. (B) Luciferase reporter assay of COL16A1 transcriptional activity with either the wild-type promoter or specific mutations (n=3). Quantification analysis of protein expression of BRG1 in (C) CTRL and DFU skin specimens, (D) wound edge of WT and db/db mice PWD 2 to 8, (E) fibroblasts cultured in LG or HG medium (n=3). (F) Quantification analysis of protein expression of BRG1 in fibroblasts transfected with Ad-NC and Ad-OE-BRG1 in HG medium (n=3). (G-N) Quantification analysis of EdU proliferation assay, Transwell migration assay, cell contraction assay and protein expression level of fibroblasts with BRG1 overexpression in HG medium (n=3). (O) Quantification analysis of protein expression of BRG1 in fibroblasts transfected with si-NC and si-BRG1 in LG medium (n=3). (P-W) Quantification analysis of EdU proliferation assay, Transwell migration assay, cell contraction assay and protein expression level of fibroblasts with BRG1 knockdown in LG medium (n=3). ns: non significance; ns: non significance; * *p* < 0.05, ** *p* < 0.01, *** *p* < 0.001, **** *p* < 0.0001. Data are expressed as mean ± SD. *p*-values were calculated by two-way ANOVA test.

**
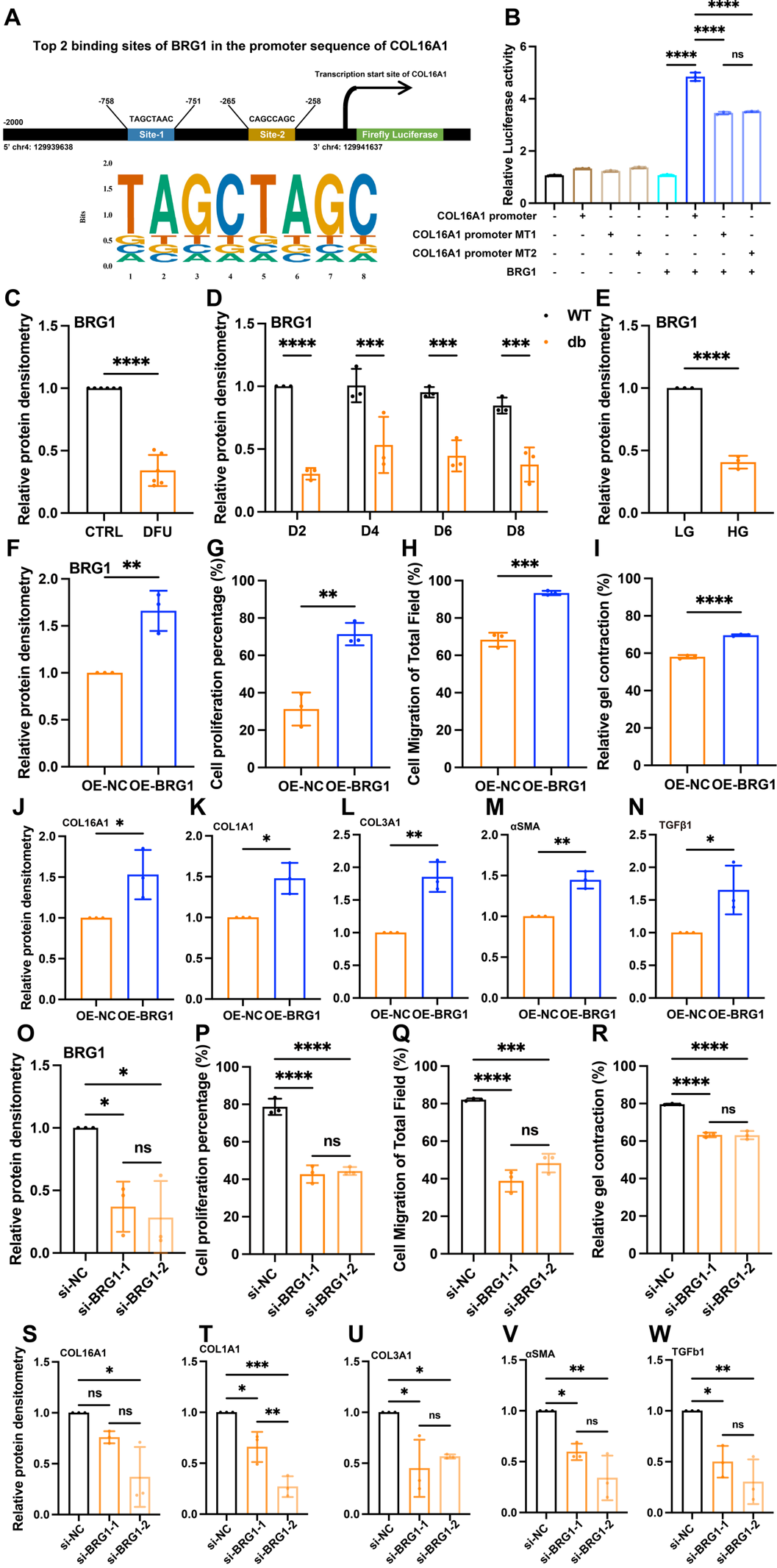
**

**Figure S7 BRG1 restores fibroblast-mediated COL16A1 secretion and promotes murine diabetic wound healing.**

(A) Representative immunofluorescent images of BRG1 (red) and DAPI (blue) in Ad-NC and Ad-OE-BRG1 transfected db/db mice PWD 2 to 8. Scale bar: 200 and 100 μm. (B-C) Representative chronological images of wounds and analysis of the wound area in Ad-NC and Ad-OE-BRG1 transfected db/db mice (n=3). (D) Trace of wound closure PWD 0 to 14. (E-F) Representative images of H&E staining and Masson staining of wounds in Ad-NC and Ad-OE-BRG1 transfected db/db mice PWD 4 to 14. (G) Immunofluorescence co-localization analysis of COL16A1 (green) and Vimentin (red) in Ad-NC and Ad-OE-BRG1 transfected db/db mice. ns: non significance; *** *p* < 0.001, **** *p* < 0.0001. Data are expressed as mean ± SD. *p*-values were calculated by the two-tailed Student’s t-test, one-way ANOVA test.

**
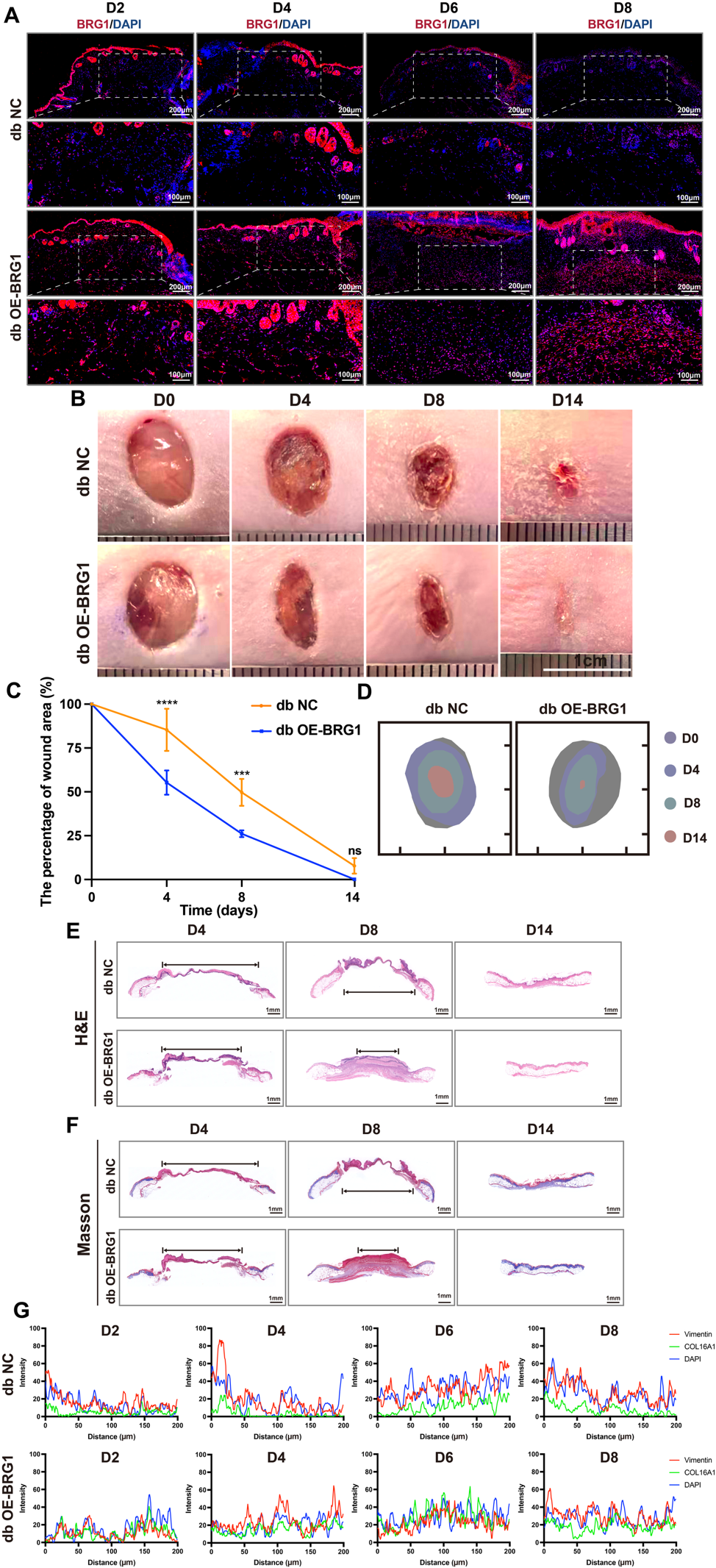
**

**Figure S8 The pro‐fibrotic effects of BRG1 depends on COL16A1.**

**
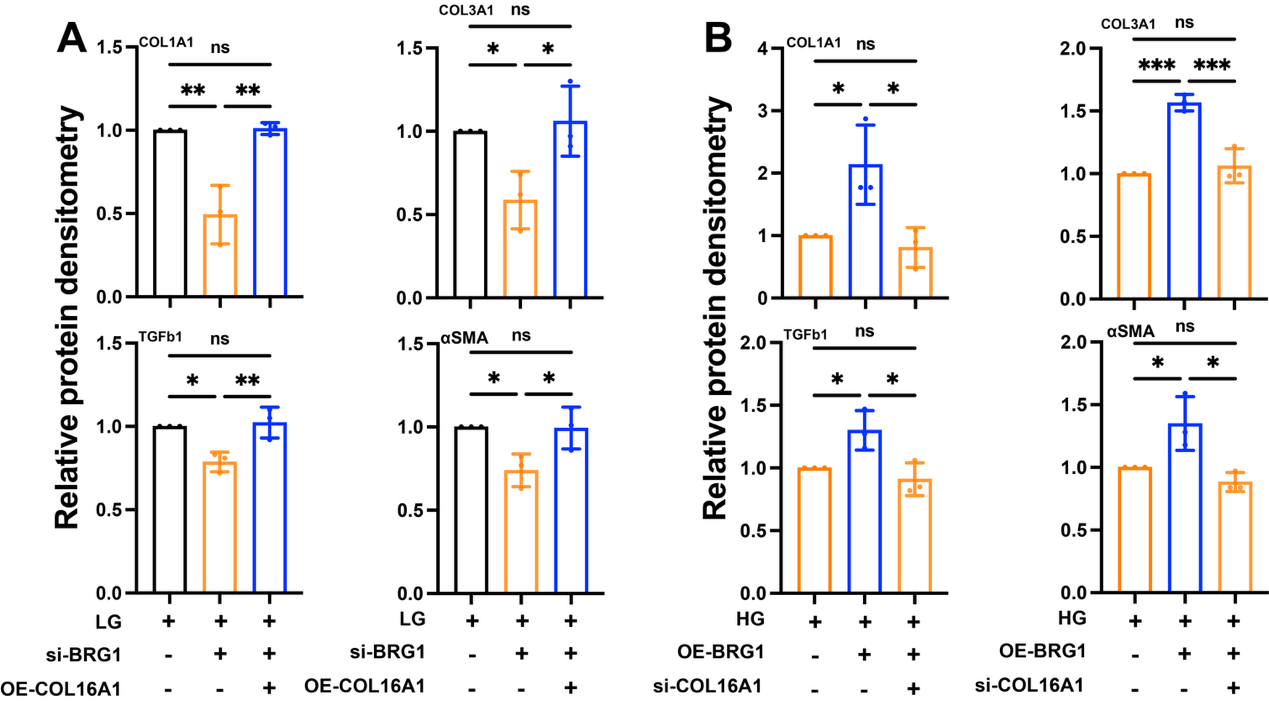
**(A) Quantification analysis of protein expression in BRG1 knockdown fibroblasts with COL16A1-overexpression in LG medium (n=3). (B) Quantification analysis of protein expression in BRG1-overexpression fibroblasts with COL16A1 knockdown in HG medium (n=3). ns: non significance; * *p* < 0.05, ** *p* < 0.01, *** *p* < 0.001. Data are expressed as mean ± SD. *p*-values were calculated by one-way ANOVA test.

**Table S1. Sequence of mouse primers used in qPCR**

| Gene symbol | Forward primer sequence (5’-3’) | Reverse primer sequence (5’-3’) |
| --- | --- | --- |
| *col16a1* | TGGGACAAATATAGGTGAGCGG | CGTTGGTTGATGGGTCACTACT |
| *brg1* | CAAAGACAAGCATATCCTAGCCA | CACGTAGTGTGTGTTAAGGACC |
| *col1a1* | GCTCCTCTTAGGGGCCACT | ATTGGGGACCCTTAGGCCAT |
| *col3a1* | CTGTAACATGGAAACTGGGGAAA | CCATAGCTGAACTGAAAACCACC |
| *tgfb1* | CCACCTGCAAGACCATCGAC | CTGGCGAGCCTTAGTTTGGAC |
| *asma* | CCCAGACATCAGGGAGTAATGG | TCTATCGGATACTTCAGCGTCA |
| *gapdh* | AGGTCGGTGTGAACGGATTTG | GGGGTCGTTGATGGCAACA |

**Table S2. Sequence of human primers used in qPCR**

| Gene symbol | Forward primer sequence (5’-3’) | Reverse primer sequence (5’-3’) |
| --- | --- | --- |
| *COL16A1* | TCGGTCTTTGGGCTACCTTC | CCCGAGGGAATACTCTTCGC |
| *GAPDH* | ACAACTTTGGTATCGTGGAAGG | GCCATCACGCCACAGTTTC |
